# Supplementary material for: Nonlinear thresholds in lipid-frailty interplay: Precision targets for severe airflow limitation in aging adults
Source: PLoS One. 2026 Apr 29;21(4):e0348083. doi: 10.1371/journal.pone.0348083 (PMC13127961; doi:10.1371/journal.pone.0348083)
Supplement: S5 Table — Multivariable logistic regression results after removing the small high‑education subgroup (n = 78, 7 SAL cases) to assess whether the main findings were driven by this unstable stratum. Estimates are presented for crude, age‑/gender‑adjusted, and fully adjusted models. (DOCX) [file pone.0348083.s007.docx]

**Supplementary Table 5** Sensitivity analysis excluding the high‑education subgroup

| **Exposure** | **Non-adjusted** | **Adjust I** | **Adjust II** |
| --- | --- | --- | --- |
| **Social**  Isolation |  |  |  |
| NO | 1 | 1 | 1 |
| YES | 1.384 (1.146 , 1.672) <0.001 | 1.240 (1.019 ~ 1.510) 0.032 | 1.187 (0.944 ~ 1.494) 0.143 |
| **VAI** | 0.972 (0.949 ~ 0.996) 0.022 | 0.968 (0.944 ~ 0.993) 0.013 | 0.976 (0.952 ~ 1.000) 0.053 |
| **AIP** | 0.544 (0.387 ~ 0.764) <0.001 | 0.557 (0.395 ~ 0.786) <0.001 | 0.578 (0.408 ~ 0.819) 0.002 |
| **NHDL** | 0.998 (0.997, 0.999) 0.002 | 0.998 (0.997, 0.999) 0.004 | 0.999 (0.997, 0.999) 0.009 |
| **Residual Cholesterol** | 0.725 (0.572 ~ 0.918) 0.008 | 0.717 (0.564 ~ 0.912) 0.007 | 0.756 (0.597 ~ 0.959) 0.021 |
| **EGFR** | 0.992 (0.987, 0.998) 0.005 | 0.997 (0.991, 1.003) 0.297 | 0.994 (0.989, 1.000) 0.063 |
| **Frailty Index** | 1.089 (1.066 ~ 1.112) <0.001 | 1.081 (1.057 ~ 1.105) <0.001 | 1.081 (1.058 ~ 1.105) <0.001 |
| **Frailty** |  |  |  |
| NO | 1 | 1 | 1 |
| YES | 1.963 (1.593 ~ 2.418) <0.001 | 1.814 (1.464 ~ 2.249) <0.001 | 1.830 (1.476 ~ 2.268) <0.001 |
| **ASM** | 0.931 (0.909 ~ 0.953) <0.001 | 0.870 (0.836 ~ 0.905) <0.001 | 0.901 (0.874 ~ 0.930) <0.001 |
| **Castelli Index I** | 0.788 (0.704 ~ 0.882) <0.001 | 0.782 (0.698 ~ 0.876) <0.001 | 0.790 (0.704 ~ 0.887) <0.001 |
| **Castelli Index II** | 0.739 (0.635 ~ 0.859) <0.001 | 0.725 (0.622 ~ 0.844) <0.001 | 0.732 (0.627 ~ 0.855) <0.001 |
| **Social Economic Status** |  |  |  |
| low | 1 | 1 | 1 |
| low-middle | 0.805 (0.652, 0.994) 0.044 | 0.836 (0.676, 1.033) 0.098 | 0.879 (0.708, 1.091) 0.243 |
| upper-middle | 0.643 (0.485, 0.852) 0.002 | 0.709 (0.532, 0.943) 0.018 | 0.800 (0.595, 1.075) 0.139 |

OR: Odds Ratio, CI: Confidence Interval

Model1: Crude

Model2: Adjust: age, gender

Model3: Adjust: location, marital_status, education, smoke, drink
