# Supplementary material for: Nonlinear thresholds in lipid-frailty interplay: Precision targets for severe airflow limitation in aging adults
Source: PLoS One. 2026 Apr 29;21(4):e0348083. doi: 10.1371/journal.pone.0348083 (PMC13127961; doi:10.1371/journal.pone.0348083)
Supplement: S6 Table — Multivariable logistic regression results after handling missing data with multiple imputation (5 imputations using the mice package). Continuous variables were imputed with predictive mean matching; categorical variables were imputed using logistic or polytomous regression. Pooled estimates are shown for crude, age‑/gender‑adjusted, and fully adjusted models. (DOCX) [file pone.0348083.s008.docx]

**Supplementary Table 6** Sensitivity analysis using multiple imputation

| **Exposure** | **Non-adjusted** | **Adjust I** | **Adjust II** |
| --- | --- | --- | --- |
| **Social**  Isolation |  |  |  |
| NO | 1 | 1 | 1 |
| YES | 1.430 (1.260 ~ 1.623) <0.001 | 1.263 (1.106 ~ 1.442) <0.001 | 1.203 (1.030 ~ 1.404) 0.020 |
| **VAI** | 0.977 (0.960 ~ 0.993) 0.006 | 0.977 (0.960 ~ 0.994) 0.009 | 0.981 (0.964 ~ 0.998) 0.026 |
| **AIP** | 0.581 (0.456 ~ 0.740) <0.001 | 0.617 (0.483 ~ 0.788) <0.001 | 0.617 (0.482 ~ 0.790) <0.001 |
| **NHDL** | 0.999 (0.998 ~ 0.999) <0.001 | 0.999 (0.998 ~ 0.999) 0.001 | 0.999 (0.998, 0.999) 0.002 |
| **Residual Cholesterol** | 0.751 (0.638 ~ 0.883) <.001 | 0.753 (0.639 ~ 0.889) <.001 | 0.778 (0.661 ~ 0.916) 0.003 |
| **EGFR** | 0.992 (0.988 ~ 0.996) <.001 | 0.998 (0.994 ~ 1.002) 0.401 | 0.996 (0.991 ~ 0.999) 0.032 |
| **Frailty Index** | 1.088 (1.073 ~ 1.103) <0.001 | 1.080 (1.064 ~ 1.096) <0.001 | 1.079 (1.063 ~ 1.094) <0.001 |
| **Frailty** |  |  |  |
| NO | 1 | 1 | 1 |
| YES | 1.886 (1.642 ~ 2.167) <0.001 | 1.729 (1.497 ~ 1.997) <0.001 | 1.717 (1.487 ~ 1.982) <0.001 |
| **ASM** | 0.943 (0.929 ~ 0.957) <0.001 | 0.886 (0.863 ~ 0.908) <0.001 | 0.918 (0.900 ~ 0.936) <0.001 |
| **Castelli Index I** | 0.824 (0.761 ~ 0.892) <0.001 | 0.821 (0.758 ~ 0.889) <0.001 | 0.824 (0.760 ~ 0.894) <0.001 |
| **Castelli Index II** | 0.804 (0.722 ~ 0.895) <0.001 | 0.793 (0.712 ~ 0.883) <0.001 | 0.796 (0.714 ~ 0.887) <0.001 |
| **Social Economic Status** |  |  |  |
| low | 1 | 1 | 1 |
| low-middle | 0.766 (0.669 ~ 0.878) <0.001 | 0.796 (0.694 ~ 0.914) 0.001 | 0.834 (0.725 ~ 0.959) 0.011 |
| upper-middle | 0.589 (0.484 ~ 0.718) <0.001 | 0.649 (0.531 ~ 0.794) <0.001 | 0.737 (0.598 ~ 0.908) 0.004 |
| high | 0.621 (0.213 ~ 1.813) 0.384 | 0.677 (0.231 ~ 1.986) 0.478 | 1.157 (0.356 ~ 3.760) 0.808 |

OR: Odds Ratio, CI: Confidence Interval

Model1: Crude

Model2: Adjust: age, gender

Model3: Adjust: location, marital_status, education, smoke, drink
