# Supplementary material for: Nonlinear thresholds in lipid-frailty interplay: Precision targets for severe airflow limitation in aging adults
Source: PLoS One. 2026 Apr 29;21(4):e0348083. doi: 10.1371/journal.pone.0348083 (PMC13127961; doi:10.1371/journal.pone.0348083)
Supplement: S7 Table — Outliers for continuous variables were defined as values below Q1 − 1.5 × IQR or above Q3 + 1.5 × IQR, calculated separately within each SAL group. This table presents the regression results after removing these outliers. (DOCX) [file pone.0348083.s009.docx]

**Supplementary Table 7** Multivariable logistic regression analyses after excluding outliers identified by the IQR method

| **Exposure** | **Non-adjusted** | **Adjust I** | **Adjust II** |
| --- | --- | --- | --- |
| **Social**  Isolation |  |  |  |
| NO | 1 | 1 | 1 |
| YES | 1.430 (1.260 ~ 1.623) <0.001 | 1.263 (1.106 ~ 1.442) <0.001 | 1.203 (1.030 ~ 1.404) 0.020 |
| **VAI** | 0.918 (0.876 ~ 0.961) <0.001 | 0.901 (0.857 ~ 0.947) <0.001 | 0.921 (0.877 ~ 0.967) <0.001 |
| **AIP** | 0.373 (0.240 ~ 0.580) <0.001 | 0.384 (0.246 ~ 0.600) <0.001 | 0.403 (0.256 ~ 0.633) <0.001 |
| **NHDL** | 0.996 (0.994 ~ 0.998) <0.001 | 0.996 (0.994 ~ 0.998) <0.001 | 0.996 (0.994 ~ 0.998) <0.001 |
| **Residual Cholesterol** | 0.518 (0.349 ~ 0.768) 0.001 | 0.498 (0.333 ~ 0.745) <0.001 | 0.561 (0.376 ~ 0.838) 0.005 |
| **EGFR** | 0.995 (0.987 ~ 1.002) 0.177 | 1.004 (0.995 ~ 1.013) 0.413 | 0.998 (0.990 ~ 1.006) 0.591 |
| **Frailty Index** | 1.174 (1.140 ~ 1.208) <0.001 | 1.165 (1.131 ~ 1.201) <0.001 | 1.164 (1.129 ~ 1.199) <0.001 |
| **Frailty** |  |  |  |
| NO | 1 | 1 | 1 |
| YES | 1.886 (1.642 ~ 2.167) <0.001 | 1.729 (1.497 ~ 1.997) <0.001 | 1.717 (1.487 ~ 1.982) <0.001 |
| **ASM** | 0.943 (0.929 ~ 0.957) <0.001 | 0.886 (0.863 ~ 0.908) <0.001 | 0.918 (0.900 ~ 0.936) <0.001 |
| **Castelli Index I** | 0.689 (0.597 ~ 0.794) <0.001 | 0.680 (0.588 ~ 0.785) <0.001 | 0.690 (0.596 ~ 0.800) <0.001 |
| **Castelli Index II** | 0.659 (0.552 ~ 0.786) <0.001 | 0.645 (0.539 ~ 0.772) <0.001 | 0.657 (0.548 ~ 0.789) <0.001 |
| **Social Economic Status** |  |  |  |
| low | 1 | 1 | 1 |
| low-middle | 0.766 (0.669 ~ 0.878) <0.001 | 0.796 (0.694 ~ 0.914) 0.001 | 0.834 (0.725 ~ 0.959) 0.011 |
| upper-middle | 0.589 (0.484 ~ 0.718) <0.001 | 0.649 (0.531 ~ 0.794) <0.001 | 0.737 (0.598 ~ 0.908) 0.004 |
| high | 0.621 (0.213 ~ 1.813) 0.384 | 0.677 (0.231 ~ 1.986) 0.478 | 1.157 (0.356 ~ 3.760) 0.808 |

OR: Odds Ratio, CI: Confidence Interval

Model1: Crude

Model2: Adjust: age, gender

Model3: Adjust: location, marital_status, education, smoke, drink
