# Supplementary material for: Nonlinear thresholds in lipid-frailty interplay: Precision targets for severe airflow limitation in aging adults
Source: PLoS One. 2026 Apr 29;21(4):e0348083. doi: 10.1371/journal.pone.0348083 (PMC13127961; doi:10.1371/journal.pone.0348083)
Supplement: S8 Table — Complete results for VAI, AIP, NHDL, residual cholesterol, eGFR, frailty index, frailty status, ASM, Castelli index I/II, and SES. Three models are shown: crude, age/gender-adjusted, and fully adjusted (age, gender, living area, marital status, education, smoking, alcohol). OR and 95% CI are reported. Corresponds to Table 2. (DOCX) [file pone.0348083.s010.docx]

**Supplementary Table 8：**Full multivariate logistic regression results for all lipid parameters, muscle indices, and socioeconomic variables

| **Exposure** | **Non-adjusted** | **Adjust I** | **Adjust II** |
| --- | --- | --- | --- |
| **Social**  Isolation |  |  |  |
| NO | 1 | 1 | 1 |
| YES | 1.420 (1.177, 1.712) <0.001 | 1.273 (1.047, 1.548) 0.015 | 1.202 (0.956, 1.511) 0.115 |
| **VAI** | 0.969 (0.946, 0.993) 0.011 | 0.965 (0.941, 0.990) 0.007 | 0.974 (0.950, 0.998) 0.033 |
| **AIP** | 0.514 (0.367, 0.720) <0.001 | 0.528 (0.375, 0.743) <0.001 | 0.556 (0.394, 0.787) <0.001 |
| **NHDL** | 0.998 (0.997, 0.999) <0.001 | 0.998 (0.997, 0.999) 0.002 | 0.998 (0.997, 1.000) 0.005 |
| **Residual Cholesterol** | 0.693 (0.548, 0.877) 0.002 | 0.686 (0.540, 0.873) 0.002 | 0.732 (0.577, 0.927) 0.010 |
| **EGFR** | 0.992 (0.987, 0.998) 0.006 | 0.997 (0.991, 1.003) 0.356 | 0.995 (0.989, 1.000) 0.068 |
| **Frailty Index** | 1.091 (1.068, 1.114) <0.001 | 1.083 (1.059, 1.107)<0.001 | 1.082 (1.058, 1.106) <0.001 |
| **Frailty** |  |  |  |
| NO | 1 | 1 | 1 |
| YES | 1.962 (1.594, 2.414)<0.001 | 1.812 (1.464, 2.243) <0.001 | 1.816 (1.467, 2.248) <0.001 |
| **ASM** | 0.929 (0.908, 0.951) <0.001 | 0.867 (0.833, 0.901) <0.001 | 0.903 (0.876, 0.932) <0.001 |
| **Castelli Index I** | 0.775 (0.693, 0.866)<0.001 | 0.770 (0.688, 0.862) <0.001 | 0.782 (0.697, 0.877) <0.001 |
| **Castelli Index II** | 0.729 (0.627, 0.848) <0.001 | 0.718 (0.617, 0.835) <0.001 | 0.729 (0.625, 0.850) <0.001 |
| **Social Economic Status** |  |  |  |
| low | 1 | 1 | 1 |
| low-middle | 0.800 (0.648, 0.987) 0.037 | 0.830 (0.672, 1.026) 0.085 | 0.883 (0.712, 1.096) 0.259 |
| upper-middle | 0.610 (0.462, 0.807) <0.001 | 0.671 (0.506, 0.891) 0.006 | 0.791 (0.588, 1.063) 0.119 |
| high | 0.489 (0.110, 2.173) 0.347 | 0.539 (0.121, 2.409) 0.419 | 1.524 (0.261, 8.885) 0.640 |

Footnote：OR, Odds Ratio; CI, Confidence Interval; VAI, Visceral Adiposity Index; AIP, Atherogenic Index of Plasma; NHDL, Non-High-Density Lipoprotein Cholesterol; RC, Residual Cholesterol; eGFR, estimated Glomerular Filtration Rate; ASM, Appendicular Skeletal Muscle Mass; SES, Socioeconomic Status. Model 1: crude (unadjusted). Model 2: adjusted for age and gender. Model 3: adjusted for age, gender, living area, marital status, education, smoking, and alcohol consumption. All lipid variables were analyzed in separate models.
