# Supplementary material for: Nonlinear thresholds in lipid-frailty interplay: Precision targets for severe airflow limitation in aging adults
Source: PLoS One. 2026 Apr 29;21(4):e0348083. doi: 10.1371/journal.pone.0348083 (PMC13127961; doi:10.1371/journal.pone.0348083)
Supplement: S9 Table — Complete threshold analysis for VAI, AIP, NHDL, residual cholesterol, eGFR, frailty index, ASM, and Castelli index I/II. For each exposure, the table shows linear effect, inflection point (K), segment-specific effects (below/above K), sample sizes per segment, and log-likelihood ratio test P-value. All analyses are fully adjusted. Corresponds to Table 3. (DOCX) [file pone.0348083.s011.docx]

**Supplementary Table 9：**Full threshold analysis results for all continuous exposures

| **exposure:** | **VAI** | **AIP** | **NHDL** | **Residual Cholesterol** | **EGFR** | **Frailty Index** | **ASM** | **Castelli Index I** | **Castelli Index II** |
| --- | --- | --- | --- | --- | --- | --- | --- | --- | --- |
| **Outcome:SAL** | **OR (95%CI) *P*value** | **OR (95%CI) *P*value** | **OR (95%CI) *P*value** | **OR (95%CI) *P*value** | **OR (95%CI) *P*value** | **OR (95%CI) *P*value** | **OR (95%CI) *P*value** | **OR (95%CI) *P*value** | **OR (95%CI) *P*value** |
| A straight line effect | 0.970 (0.945, 0.994) 0.017 | 0.556 (0.392, 0.788) 0.001 | 0.998 (0.997, 1.000) 0.006 | 0.716 (0.562, 0.912) 0.007 | 0.997 (0.991, 1.003) 0.341 | 1.077 (1.053, 1.101) <0.001 | 0.881 (0.846, 0.917) <0.001 | 0.774 (0.690, 0.869) <0.001 | 0.717 (0.615, 0.838) <0.001 |
| Model II |  |  |  |  |  |  |  |  |  |
| Folding point (K) | 4.687 | 0.661 | 57.724 | 0.329 | 58.308 | 2.571 | 23.917 | 3.567 | 1.791 |
| n below K / n above K | 1844/1063 | 2450/457 | 1343/1564 | 130/2777 | 156/2751 | 787/2120 | 2758/149 | 1386/1521 | 1057/1850 |
| <K-segment effect 1 | 0.891 (0.820, 0.967) 0.006 | 0.476 (0.304, 0.743) 0.001 | 0.994 (0.990, 0.999) 0.008 | 0.003 (0.000, 0.473) 0.024 | 0.981 (0.959, 1.003) 0.088 | 1.299 (1.054, 1.600) 0.014 | 0.872 (0.835, 0.909) <0.001 | 0.582 (0.457, 0.742) <0.001 | 0.498 (0.336, 0.737) <0.001 |
| >K-segment effect 2 | 0.991 (0.961, 1.022) 0.5577 | 1.083 (0.320, 3.661) 0.8984 | 0.999 (0.998, 1.001) 0.3063 | 0.758 (0.595, 0.967) 0.0257 | 1.001 (0.993, 1.009) 0.8397 | 1.066 (1.040, 1.093) <0.0001 | 1.044 (0.862, 1.265) 0.6568 | 0.927 (0.793, 1.084) 0.3414 | 0.847 (0.682, 1.051) 0.1316 |
| Log Likelihood Ratio Tests | 0.035 | 0.276 | 0.049 | 0.039 | 0.143 | 0.069 | 0.131 | 0.011 | 0.050 |

Footnote：VAI, Visceral Adiposity Index; AIP, Atherogenic Index of Plasma; NHDL, Non-High-Density Lipoprotein Cholesterol; RC, Residual Cholesterol; eGFR, estimated Glomerular Filtration Rate; ASM, Appendicular Skeletal Muscle Mass; K, inflection point (threshold); LRT, Likelihood Ratio Test for nonlinearity. ORs and 95% CIs are presented for linear effects and for segments below (<K) and above (>K) the estimated threshold. All analyses were adjusted for age, gender, living area, marital status, education, smoking, and alcohol consumption.n below K and n above K indicate the number of participants in each segment of the piecewise regression model for variables with significant nonlinearity.
