# Supplementary material for: Nonlinear thresholds in lipid-frailty interplay: Precision targets for severe airflow limitation in aging adults
Source: PLoS One. 2026 Apr 29;21(4):e0348083. doi: 10.1371/journal.pone.0348083 (PMC13127961; doi:10.1371/journal.pone.0348083)
Supplement: S10 Table — Complete interaction results for all exposures (VAI, AIP, NHDL, residual cholesterol, eGFR, frailty index, ASM, Castelli index I/II) across three education strata. OR and 95% CI are shown for crude, age/gender-adjusted, and fully adjusted models, along with P-value for interaction. Sample sizes: no formal education (n = 1,279; SAL = 298), high school and below (n = 1,550; SAL = 251), above high school (n = 78; SAL = 7). Corresponds to Table 4. (DOCX) [file pone.0348083.s012.docx]

**Supplementary Table 10：**Full interaction analysis results for all exposures stratified by education level

| **Model** | **No formal education** | **High school and below** | **Above high school** | ***P* interaction** |
| --- | --- | --- | --- | --- |
| **Outcome:SAL** | **OR (95%CI) *P*value** | **OR (95%CI) *P*value** | **OR (95%CI) *P*value** |  |
| **VAI** |  |  |  |  |
| Crude | 0.991 (0.962, 1.021) 0.558 | 0.943 (0.905, 0.982) 0.004 | 0.678 (0.441, 1.042) 0.076 | 0.019 (0.015 #) |
| Model I* | 0.994 (0.964, 1.026) 0.713 | 0.936 (0.897, 0.977) 0.003 | 0.719 (0.467, 1.108) 0.135 | 0.019 (0.009 #) |
| Model II* | 0.997 (0.967, 1.027) 0.823 | 0.946 (0.907, 0.986) 0.009 | 0.617 (0.366, 1.039) 0.069 | 0.012 (0.013 #) |
| **AIP** |  |  |  |  |
| Crude | 0.745 (0.465, 1.194) 0.221 | 0.428 (0.261, 0.704) <0.001 | 0.031 (0.001, 0.957) 0.047 | 0.057 (0.038 #) |
| Model I* | 0.766 (0.476, 1.233) 0.273 | 0.433 (0.262, 0.716) 0.001 | 0.042 (0.001, 1.255) 0.067 | 0.071 (0.039 #) |
| Model II* | 0.773 (0.479, 1.248) 0.292 | 0.427 (0.257, 0.710) 0.001 | 0.015 (0.000, 0.855) 0.042 | 0.036 (0.029 #) |
| **NHDL** |  |  |  |  |
| Crude | 0.999 (0.998, 1.001) 0.343 | 0.997 (0.996, 0.999) 0.002 | 0.986 (0.970, 1.002) 0.080 | 0.043 (0.033 #) |
| Model I* | 0.999 (0.998, 1.001) 0.423 | 0.997 (0.996, 0.999) 0.003 | 0.987 (0.971, 1.003) 0.100 | 0.051 (0.033 #) |
| Model II* | 0.999 (0.998, 1.001) 0.490 | 0.997 (0.996, 0.999) 0.003 | 0.984 (0.966, 1.002) 0.080 | 0.031 (0.027 #) |
| **Residual Cholesterol** |  |  |  |  |
| Crude | 0.876 (0.644, 1.191) 0.398 | 0.580 (0.400, 0.841) 0.004 | 0.010 (0.000, 0.678) 0.032 | 0.009 (0.019 #) |
| Model I* | 0.893 (0.655, 1.218) 0.476 | 0.571 (0.391, 0.833) 0.004 | 0.013 (0.000, 0.872) 0.043 | 0.012 (0.017 #) |
| Model II* | 0.908 (0.667, 1.236) 0.539 | 0.599 (0.412, 0.870) 0.007 | 0.009 (0.000, 0.709) 0.035 | 0.009 (0.020 #) |
| **EGFR** |  |  |  |  |
| Crude | 0.996 (0.988, 1.003) 0.252 | 0.991 (0.983, 0.999) 0.028 | 1.010 (0.954, 1.070) 0.725 | 0.600 (0.539 #) |
| Model I* | 0.997 (0.989, 1.005) 0.474 | 0.997 (0.988, 1.006) 0.529 | 1.025 (0.957, 1.097) 0.480 | 0.723 (0.811 #) |
| Model II* | 0.997 (0.989, 1.005) 0.405 | 0.992 (0.984, 1.000) 0.060 | 1.013 (0.951, 1.078) 0.690 | 0.617 (0.552 #) |
| **Frailty Index** |  |  |  |  |
| Crude | 1.085 (1.055, 1.116) <0.001 | 1.073 (1.037, 1.111) <0.001 | 1.182 (0.940, 1.485) 0.152 | 0.666 (0.797 #) |
| Model I* | 1.088 (1.057, 1.120) <0.001 | 1.059 (1.022, 1.097) 0.002 | 1.181 (0.924, 1.509) 0.183 | 0.385 (0.355 #) |
| Model II* | 1.087 (1.056, 1.118) <0.001 | 1.073 (1.036, 1.111) <0.001 | 1.192 (0.937, 1.516) 0.152 | 0.629 (0.758 #) |
| **ASM** |  |  |  |  |
| Crude | 0.951 (0.918, 0.985) 0.005 | 0.934 (0.901, 0.968) 0.002 | 0.974 (0.826, 1.149) 0.757 | 0.723 (0.618 #) |
| Model I* | 0.866 (0.817, 0.918) <0.001 | 0.886 (0.838, 0.937) <0.001 | 0.810 (0.566, 1.158) 0.248 | 0.776 (0.716 #) |
| Model II* | 0.914 (0.875, 0.954) <0.001 | 0.889 (0.851, 0.930) <0.001 | 0.984 (0.815, 1.187) 0.863 | 0.493 (0.648 #) |
| **Castelli Index I** |  |  |  |  |
| Crude | 0.863 (0.740, 1.006) 0.060 | 0.732 (0.621, 0.864) <0.001 | 0.379 (0.130, 1.105) 0.076 | 0.125 (0.067 #) |
| Model I* | 0.865 (0.741, 1.010) 0.067 | 0.723 (0.612, 0.854) <0.001 | 0.402 (0.125, 1.294) 0.127 | 0.143 (0.064 #) |
| Model II* | 0.855 (0.730, 1.001) 0.051 | 0.728 (0.615, 0.862) <0.001 | 0.334 (0.105, 1.065) 0.064 | 0.116 (0.073 #) |
| **Castelli Index** **II** |  |  |  |  |
| Crude | 0.790 (0.639, 0.976) 0.029 | 0.707 (0.568, 0.880) 0.002 | 0.501 (0.133, 1.883) 0.306 | 0.649 (0.384 #) |
| Model I* | 0.788 (0.637, 0.974) 0.028 | 0.691 (0.555, 0.860) <0.001 | 0.609 (0.154, 2.407) 0.479 | 0.674 (0.374 #) |
| Model II* | 0.771 (0.621, 0.958) 0.019 | 0.698 (0.559, 0.872) 0.002 | 0.434 (0.105, 1.783) 0.247 | 0.624 (0.405 #) |

Model I has been adjusted:age, gender and the interaction terms for following variables: age, gender.

Model II has been adjusted:living area, marital status, smoke status, drinking status and the interaction terms for following variables: living area, marital status, smoke status, drinking status.

Sample sizes: No formal education (n=1,279; SAL=298), High school and below (n=1,550; SAL=251), Above high school (n=78; SAL=7).Cell size < 30 participants in the above‑high‑school stratum (n = 78 total; 7 SAL cases); estimates may be unstable and should be interpreted with caution. These findings are exploratory and hypothesis‑generating only, and may not be replicable in larger samples.

| **Frailty Index** | | | |
| --- | --- | --- | --- |
| **Outcome:SAL** | **OR (95%CI) *P*value** | **OR (95%CI) *P*value** | ***P* interaction** |
| Crude | 1.138 (1.088, 1.190) <0.001 | 1.075 (1.050, 1.101) <0.001 | 0.028 |
| Model I* | 1.132 (1.081, 1.186) <0.001 | 1.067 (1.041, 1.095) <0.001 | 0.028 |
| Model II* | 1.135 (1.083, 1.189) <0.001 | 1.068 (1.042, 1.095) <0.001 | 0.024 |

Model I has been adjusted:age, gender and the interaction terms for following variables: age, gender.

Model II has been adjusted:living area, marital status, smoke status, drinking status and the interaction terms for following variables: living area, marital status, smoke status, drinking status.
