# Supplementary material for: Nonlinear thresholds in lipid-frailty interplay: Precision targets for severe airflow limitation in aging adults
Source: PLoS One. 2026 Apr 29;21(4):e0348083. doi: 10.1371/journal.pone.0348083 (PMC13127961; doi:10.1371/journal.pone.0348083)
Supplement: S2 File — This file contains the authors’ competing interests declaration. (DOCX) [file pone.0348083.s016.docx]

**Declaration of Interest Statement**

The authors declare no competing financial interests or personal relationships that could influence the work reported in this paper. This study received no specific grant from funding agencies in the public, commercial, or not-for-profit sectors. All authors confirm independence in designing the study, interpreting data, writing the manuscript, and deciding to submit for publication.
