# Supplementary material for: Nonlinear thresholds in lipid-frailty interplay: Precision targets for severe airflow limitation in aging adults
Source: PLoS One. 2026 Apr 29;21(4):e0348083. doi: 10.1371/journal.pone.0348083 (PMC13127961; doi:10.1371/journal.pone.0348083)
Supplement: Attachment — Submitted filename: PONE -D-25-40637_R2 Reviewer Recommendation-Accept.pdf [file pone.0348083.s021.pdf]

Reviewer Recommendation: Accept with Minor Editorial Suggestions

The authors have made substantial improvements to the manuscript and have addressed the major concerns raised in the earlier rounds of review. In particular, the interpretation of findings has been appropriately moderated, with careful consideration of reverse causation and the lipid paradox. The subgroup and interaction analyses are now presented with adequate caution, and the handling of missing data is clearly described. Overall, the study is scientifically sound and suitable for publication.

I would recommend acceptance of the manuscript, subject to a few minor editorial refinements:

1. Figures (Figures 2–3):
   The inclusion of forest plots is a welcome improvement. However, the figures remain somewhat dense and may benefit from simplification. Reducing the number of variables per panel and improving label clarity would enhance readability for a general audience.
2. Methods clarity:
   While improved, a brief clarification of the inclusion/exclusion criteria and more explicit description of the threshold modeling approach (e.g., segmentation details) would further strengthen transparency.
3. Tables and presentation:
   Some tables remain slightly difficult to interpret, and minor formatting adjustments may improve clarity.
4. Language and editorial aspects:
   A final round of language editing is recommended to address minor grammatical and stylistic inconsistencies.

In summary, the manuscript is of good quality, and I support its publication after these minor revisions.
