# Supplementary material for: Nonlinear thresholds in lipid-frailty interplay: Precision targets for severe airflow limitation in aging adults
Source: PLoS One. 2026 Apr 29;21(4):e0348083. doi: 10.1371/journal.pone.0348083 (PMC13127961; doi:10.1371/journal.pone.0348083)
Supplement: Attachment — Submitted filename: Response to Reviewers.docx [file pone.0348083.s020.docx]

**Point-by-point Response Letter-[Manuscript ID:PONE-D-25-40637]**

Original Title:**Nonlinear Thresholds in Lipid-Frailty Interplay: Precision Targets for Pulmonary Flow Restriction in Aging Adults**

Revised title:**Nonlinear Thresholds in Lipid-Frailty Interplay: Precision Targets for Severe Airflow Limitation in Aging Adults**

**Dear Editors and Reviewers of Plos One,**

We are deeply grateful for considering our manuscript for ***Plos One*** and value the insightful comments offered. These suggestions have been crucial for enhancing our manuscript's quality, and we're confident the revisions have significantly improved its clarity, rigor, and impact. We've meticulously revised the manuscript, addressing each comment with careful consideration and research. Besides responding to the feedback, we've also thoroughly reviewed the entire manuscript, making additional refinements to ensure top - notch scientific and literary quality. All changes are clearly tracked in the revised manuscript, and a clean version is also prepared. Below are our detailed point-by-point responses to each comment. We've aimed to be comprehensive and transparent in our explanations, providing evidence and reasoning for the changes made. Thank you again sincerely for your dedication and hard work. Your expertise is invaluable to the scientific community, and we're honored to contribute to this prestigious journal.

Yours Sincerely,

ZhongQiang Guo

Henan University

**[guozhongqiang0701@gmail.com](mailto:guozhongqiang0701@gmail.com)**

**Editor**

**Comments/suggestions:
When submitting your revision, we need you to address these additional requirements.**

**1. Please ensure that your manuscript meets PLOS ONE's style requirements, including those for file naming. The PLOS ONE style templates can be found at**

**<https://journals.plos.org/plosone/s/file?id=wjVg/PLOSOne_formatting_sample_main_body.pdf> and <https://journals.plos.org/plosone/s/file?id=ba62/PLOSOne_formatting_sample_title_authors_affiliations.pdf>**

**Response:**

We would like to express our sincere gratitude for giving us the opportunity to revise and resubmit our manuscript. We truly appreciate the time and effort you and the reviewers have dedicated to providing such thoughtful and constructive feedback. Your guidance has been invaluable in helping us improve the clarity and rigor of our work, and we are very grateful for the chance to address the comments and strengthen the manuscript.

Thank you for providing the detailed formatting guidelines. We have carefully reviewed the PLOS ONE style templates and have revised our manuscript to ensure full compliance. The following adjustments have been made:

1. Manuscript Body Formatting:

The entire manuscript has been reformatted to double-space, as per the guidelines.

All major section headings (Abstract, Introduction, Materials and methods, Results, Discussion, etc.) are now formatted as Level 1 headings (Bold, 18pt, sentence case).

Sub-sections within major sections now use Level 2 headings (Bold, 16pt, sentence case).

All in-text citations for figures, tables, supporting information, and references have been checked to ensure they follow the required formats (e.g., "Fig 1", "Table 1", "S1 Table", "[1]").

2. Figure and Table Placement:

All figures have been removed from the manuscript file and are now uploaded separately as individual files, named according to the specifications (e.g., Fig1.tif, Fig2.tif).

Figure captions have been placed directly after the paragraph in which the figure is first cited in the manuscript text.

Tables are included directly after the paragraph in which they are first cited and are formatted as cell-based tables.

3. File Naming:

Main Manuscript: Manuscript.docx

igures: Fig1.tif, Fig2.tif, Fig3.tif (and so on, for all main figures).

Supporting Information: All supporting files have been renamed to match the conventions in the captions list at the end of the manuscript (e.g., S1_Table.xlsx, S2_Appendix.docx, S1_Code.R). A complete list is provided in the "Supporting information" section of the manuscript.

4. Reference List:

The reference list has been formatted according to the PLOS ONE style, ensuring that all entries with more than six authors list the first six names followed by "et al."

We believe the manuscript now fully meets PLOS ONE's formatting requirements. Thank you for your guidance.

**2. Please include your full and correct ethics statement in the “Methods” section of your manuscript file. In your statement, please include the full name of the IRB or ethics committee who approved or waived your study, as well as whether or not you obtained informed written or verbal consent. If consent was waived for your study, please include this information in your statement as well. If your ethics statement is written in any section besides the Methods, please delete it from any other section.**

**Response:**

Thank you for your detailed guidance regarding the ethics statement. We have carefully revised the manuscript to ensure full compliance with your requirements.

Response:

We have updated the ethics statement in the Materials and methods section to include all required information. The revised statement now reads:

“Study Population

This study is based on publicly available data from the China Health and Retirement Longitudinal Study (CHARLS) database. The final sample size included in the analysis was 2907 participants, including 2351 in the group without Severe Airflow Limitation and 556 in the group with Severe Airflow Limitation. The study detailed the baseline demographic characteristics of participants (such as age, gender, place of residence, marital status, education level) and lifestyle information (such as smoking history, frequency of alcohol consumption)(16).All analyses were conducted based on the available variables in the database.This study used publicly available data from the 2015 nationwide follow-up wave of the China Health and Retirement Longitudinal Study (CHARLS).The data was obtained through authorization from the CHARLS Data Center of Peking University on May 19, 2025.The original CHARLS study was approved by the Biomedical Ethics Review Committee of Peking University (IRB00001052-1015). All participants in the original CHARLS survey provided written informed consent prior to data collection. For this secondary analysis, all data were fully anonymized and de-identified prior to receipt by the authors. As this study used only publicly available, de-identified data from the CHARLS database, no additional ethical approval or informed consent was required(17, 18)(Figure 1).”
We have ensured that:

Full IRB name is included: Biomedical Ethics Review Committee of Peking University

IRB approval number is provided: *IRB00001052-1015*

Consent information is clearly stated: written informed consent was obtained from all original CHARLS participants

Waiver statement is included: no additional ethical approval or consent was required for this secondary analysis due to the use of fully anonymized, publicly available data

The ethics statement appears exclusively in the Methods section and has been removed from any other sections

We confirm that the revised manuscript meets PLOS ONE's ethical reporting requirements.

Thank you again for your valuable guidance.

**3. Please upload a new copy of Figure 2 as the detail is not clear. Please follow the link for more information:  <https://journals.plos.org/plosone/s/figures>**

**Response:**

**4. Please remove your figures from within your manuscript file, leaving only the individual TIFF/EPS image files, uploaded separately. These will be automatically included in the reviewers’ PDF.**

**Response:**

Thank you for your suggestion. We have resized the images and adjusted the font to ensure all details are clear and meet the requirements of the journal.

Thank you again for your guidance.

**5.Please include captions for your Supporting Information files at the end of your manuscript, and update any in-text citations to match accordingly. Please see our Supporting Information guidelines for more information: <http://journals.plos.org/plosone/s/supporting-information>.**

**Response:**

Thank you for your guidance regarding the Supporting Information. We have carefully reviewed the PLOS ONE Supporting Information guidelines and have revised the manuscript accordingly.

We have added a complete list of captions for all Supporting Information files at the end of the manuscript, under a Level 1 heading "Supporting information". Each caption follows the required format: file number, file name, and a brief description. We have also updated all in-text citations to match the file names.

Specifically:

Captions added at the end of the manuscript (after the References section):

S1 Data. Raw data.The raw data mentioned in this article are all located in this file.

S1 File. README.The data interpretation of the raw data mentioned in this article is located in this file.

S2 Supplementary Table 1. FDR‑adjusted results for multivariate logistic regression analyses. This table presents the raw p‑values and False Discovery Rate (FDR)‑adjusted q‑values for all associations shown in Table 2 (main text). Variables with q < 0.05 are considered significant after multiple testing correction.

S2 Supplementary Table 2. FDR‑adjusted results for threshold effect analyses. This table provides the raw p‑values and FDR‑adjusted q‑values for the linear and segmented regression results shown in Table 3. The log‑likelihood ratio test (LRT) for nonlinearity is also included. Significant nonlinearity is indicated by q < 0.05; suggestive nonlinearity (p < 0.05 but q ≥ 0.05) is noted.

S2 Supplementary Table 3. FDR‑adjusted results for interaction analyses. This table reports the raw p‑values and FDR‑adjusted q‑values for the interaction effects between education level and each exposure variable, corresponding to Table 4. Estimates for the three education strata are also provided with their adjusted significance status.

S2 Supplementary Table 4. Bootstrap validation of threshold estimates. Threshold estimates (K) for each variable with a nonlinear association were validated using 1,000 bootstrap iterations. The table shows the linear effect, the original K, the segment‑specific odds ratios, the likelihood ratio test p‑value, the bootstrap mean K, and the 95% confidence interval of K.

S2 Supplementary Table 5. Sensitivity analysis excluding the high‑education subgroup. Multivariable logistic regression results after removing the small high‑education subgroup (n = 78, 7 SAL cases) to assess whether the main findings were driven by this unstable stratum. Estimates are presented for crude, age‑/gender‑adjusted, and fully adjusted models.

S2 Supplementary Table 6. Sensitivity analysis using multiple imputation. Multivariable logistic regression results after handling missing data with multiple imputation (5 imputations using the mice package). Continuous variables were imputed with predictive mean matching; categorical variables were imputed using logistic or polytomous regression. Pooled estimates are shown for crude, age‑/gender‑adjusted, and fully adjusted models.

S2 Supplementary Table 7. Multivariable logistic regression analyses after excluding outliers identified by the IQR method. Outliers for continuous variables were defined as values below Q1 − 1.5×IQR or above Q3 + 1.5×IQR, calculated separately within each SAL group. This table presents the regression results after removing these outliers.

S3 Supplementary Code 1. R code for data processing and outlier removal. This script reads the raw data, performs variable cleaning, and removes outliers for continuous variables using the interquartile range (IQR) method within each SAL group. The cleaned dataset is saved for subsequent analyses.

S3 Supplementary Code 2. R code for multiple imputation. This script uses the mice package to perform multiple imputation (m = 5) for missing values. Continuous variables are imputed with predictive mean matching; categorical variables are imputed using appropriate methods. The five imputed datasets are combined by averaging continuous variables and taking the mode for categorical variables. The final complete dataset is exported.

S3 Supplementary Code 3. R code for VIF calculation and FDR correction. This script calculates variance inflation factors (VIF) for the lipid metabolism variables to assess multicollinearity. It also performs False Discovery Rate (Benjamini–Hochberg) correction for the p‑values from the interaction analyses (Table 4) and the threshold effect analyses (Table 3). Corrected q‑values and significance flags are output.

File naming: All Supporting Information files have been renamed to match the captions (e.g., "S1_Data.xlsx", "S1_File.pdf", "S2_Table.docx", "S1_Code.R", etc.).

In-text citations: We have verified that all in-text citations to Supporting Information files (e.g., "Supplementary Table 1", "Supplementary Code 1") have been updated to the standard format (e.g., "S1 Table", "S1 Code") and correctly match the file names and captions.

We confirm that the Supporting Information section now fully complies with PLOS ONE guidelines.

Thank you again for your valuable guidance.

**6. We note that there is identifying data in the Supporting Information file <raw data.xlsx>. Due to the inclusion of these potentially identifying data, we have removed this file from your file inventory. Prior to sharing human research participant data, authors should consult with an ethics committee to ensure data are shared in accordance with participant consent and all applicable local laws.**

**Data sharing should never compromise participant privacy. It is therefore not appropriate to publicly share personally identifiable data on human research participants. The following are examples of data that should not be shared:**

**-Name, initials, physical address
-Ages more specific than whole numbers
-Internet protocol (IP) address
-Specific dates (birth dates, death dates, examination dates, etc.)
-Contact information such as phone number or email address
-Location data
-ID numbers that seem specific (long numbers, include initials, titled “Hospital ID”) rather than random (small numbers in numerical order)**

**Data that are not directly identifying may also be inappropriate to share, as in combination they can become identifying. For example, data collected from a small group of participants, vulnerable populations, or private groups should not be shared if they involve indirect identifiers (such as sex, ethnicity, location, etc.) that may risk the identification of study participants.**

**Additional guidance on preparing raw data for publication can be found in our Data Policy (https://journals.plos.org/plosone/s/data-availability#loc-human-research-participant-data-and-other-sensitive-data) and in the following article: http://www.bmj.com/content/340/bmj.c181.long.**

**Please remove or anonymize all personal information (ID numbers), ensure that the data shared are in accordance with participant consent, and re-upload a fully anonymized data set. Please note that spreadsheet columns with personal information must be removed and not hidden as all hidden columns will appear in the published file.**

**Response:**

Thank you for your detailed guidance regarding the raw data file and for providing the additional resources on data anonymization. We fully understand the importance of protecting participant privacy and have taken immediate steps to address this issue.

We have removed all identifiable information from the raw data.To assist with data interpretation, we have prepared a README file (S1 File)

We confirm that the shared data are now in full compliance with PLOS ONE's data policy and with the ethical requirements for protecting participant privacy. The dataset contains no personally identifiable information, either directly or indirectly.

Thank you again for your careful oversight and for providing the additional resources to guide us through this process.

**Reviewer 1**

**Comments/suggestions:**

**This cross sectional analysis claims nonlinear thresholds linking lipid indices, frailty metrics, and what the authors call severe pulmonary blood flow restriction in middle aged and older adults from CHARLS. Although the topic is important, the manuscript contains serious conceptual mistakes, contradictory statements, non standard outcome naming, unstable subgroup effects, and statistical and reporting deficiencies. In its current form the work is not technically sound or interpretable. I recommend rejection. The points below explain the major reasons.**
**1.The paper repeatedly describes the outcome as severe pulmonary blood flow restriction while it is actually defined using PEF percent predicted less than 60 percent. PEF is an expiratory airflow measure, not a pulmonary blood flow metric. Conflating airway flow with blood flow is a fundamental error that undermines the title, the Abstract, and the entire interpretation. The outcome should be named severe airflow limitation and the narrative must be rewritten accordingly.**

**Response:**We thank the reviewer for identifying this critical conceptual error. We fully acknowledge that our original term, "Severe Pulmonary Flow Restriction (SPFR)," is incorrect and misleading, as the outcome is based on Peak Expiratory Flow (PEF)—a measure of expiratory airflow, not pulmonary blood flow. We have rectified this issue throughout the manuscript.

1.Outcome Renamed: We have replaced all instances of "Severe Pulmonary Flow Restriction (SPFR)" with "Severe Airflow Limitation (SAL)" . This term accurately reflects the measurement method (PEF% predicted < 60%) and aligns with standard respiratory physiology nomenclature.

2.Manuscript-wide Revision: The correction has been applied to:

①Title: Now reads: "Nonlinear Thresholds in Lipid-Frailty Interplay: Precision Targets for ②Severe Airflow Limitation in Aging Adults"

③Short Title: Now reads: "Lipid-Frailty Interplay in Airflow Limitation"

④Abstract: All instances of SPFR have been replaced with SAL.

⑤Main Text: The outcome variable is now consistently referred to as SAL throughout the Introduction, Methods, Results, Discussion, and Conclusion.

⑥Tables and Figures: All labels and captions have been updated from SPFR to SAL.

3.Conceptual Realignment: To ensure consistency with the corrected terminology, we have removed all speculative references to "pulmonary vascular damage," "blood flow restriction," and "pulmonary vasodilation" from the Introduction and Discussion. The narrative now appropriately focuses on airflow limitation, airway function, and respiratory mechanics, which are directly relevant to the PEF-based outcome.

We believe this revision resolves the fundamental conceptual error and greatly improves the clarity and accuracy of the manuscript.

**2.The Results and tables show lower AIP, lower non HDL, and lower residual cholesterol in the SPFR group, with odds ratios less than 1 that the text interprets as protection. Yet the Conclusion states that abnormal lipid profile defined as low AIP and residual cholesterol less than 0.329 and frailty are independent risk factors for SPFR. These statements are logically incompatible. The paper cannot claim both protection and risk for the same direction of change.**

**Response:**We thank the reviewer for identifying this critical logical inconsistency in our original manuscript. We fully acknowledge that our initial interpretation—claiming both "protective effects" (OR<1) and "independent risk factors" for the same lipid parameters—was contradictory and scientifically flawed.

**Explanation of the apparent paradox:**

The observation that the SAL group has significantly lower lipid levels (AIP: 0.340 vs 0.392; RC: 0.727 vs 0.792; NHDL: 77.5 vs 92.5) while regression shows OR<1 does NOT indicate that lower lipids are "protective." Rather, this pattern strongly suggests reverse causation: chronic lung disease (SAL) leads to systemic inflammation, weight loss, cachexia, and subsequent decline in lipid levels—a well-documented phenomenon in the COPD literature. The lower lipids are a consequence of the disease, not a protective factor against it. The statistical association (OR<1) merely reflects this cross-sectional relationship and cannot be interpreted causally.

**Revisions made to address this issue:**

Systematic language revision throughout the manuscript: We have replaced all causal language ("protective effect," "conferred protection," "exerted protection") with neutral descriptive terms ("inverse association," "were associated with lower odds," "showed significant associations"). This revision has been applied consistently across the Abstract, Results (including Threshold, Subgroup, and Interaction analyses), Discussion, and Conclusion.

Added explicit discussion of reverse causation to the Discussion, acknowledging that the lower lipids observed in SAL patients likely reflect disease-related metabolic changes (cachexia) rather than protective effects, and emphasizing the need for longitudinal studies to establish causality.

Revised the Conclusion to accurately reflect the findings: lipid parameters showed nonlinear associations with SAL, with lower levels observed in affected individuals—findings that likely reflect disease-related metabolic changes rather than causal protective effects. The modification of these associations by education level suggests that social factors may inform targeted intervention strategies.

Added appropriate caveats in the Results section noting that, given the cross-sectional design, these associations should not be interpreted as causal.

We believe these revisions resolve the logical inconsistency and provide a more accurate, scientifically rigorous interpretation of the data. The manuscript now clearly distinguishes between statistical associations and causal claims, and explicitly acknowledges reverse causation as the most plausible explanation for the observed lipid-SAL relationships.

**3.AIP requires triglycerides and HDL in molar units. Units are not stated consistently and the Discussion references mg per dL for residual cholesterol while tables show values that only make sense in mmol per L. PEF prediction equations are given without proper unit declarations or appropriate source verification for this population. The manuscript needs a single clear unit convention throughout and references that match the exact formulas implemented.
Response:**

We thank the reviewer for this important methodological critique regarding unit consistency and source verification. We have carefully addressed all these issues as follows:

**Unit standardization:**

We have established a single, clear unit convention throughout the manuscript. All lipid variables (triglycerides, HDL-C, LDL-C, total cholesterol, non-HDL cholesterol, and residual cholesterol) are now explicitly reported in mmol/L, which is consistent with the CHARLS data format and ensures correct calculation of derived indices. This standardization has been applied to the Abstract, Methods, Results (including all tables), Discussion, and Conclusion.

**Specifically:**

The residual cholesterol threshold previously reported as "0.329" is now clearly specified as "0.329 mmol/L" throughout the manuscript, including in the Abstract, Results section, and Table 3.

All lipid values in Table 1 (baseline characteristics) are presented in mmol/L, with units indicated in the table footnotes.

The Discussion section has been checked and revised to ensure all references to lipid values use mmol/L consistently; any instances of "mg/dL" have been corrected to "mmol/L."

**AIP calculation verification:**

The AIP formula is correctly specified as AIP = log₁₀(triglycerides/HDL-C). In the Methods section, we have explicitly stated that both triglycerides and HDL-C are in mmol/L, ensuring that the ratio is calculated using molar units as required. A citation to the original methodological literature has been included.

PEF prediction equations:

We have added explicit unit declarations to the PEF prediction equations in the Methods section:

Height: centimeters (cm)

Age: years

PEF: liters per minute (L/min)

Additionally, we have verified the source of these equations and added the appropriate citation: Nunn AJ, Gregg I. New regression equations for predicting peak expiratory flow in adults. BMJ. 1989;298(6679):1068-70. These equations have been validated in adult populations and are appropriate for use in our Chinese cohort, though we acknowledge in the Limitations that they may not be perfectly calibrated for all ethnic groups.

**Source verification for all constructed variables:**

We have reviewed and verified the primary sources for all derived indices, ensuring that each formula matches the exact implementation in our analysis and that appropriate references are cited.

We believe these revisions ensure complete clarity regarding units and sources, and we thank the reviewer for helping us improve the methodological transparency of our manuscript.

**4.The paper references the Fried phenotype while using a deficit accumulation frailty index formula. These are different frameworks. The ASM equation is presented with ambiguous gender coding and constants. Castelli indices are misspelled and not clearly derived from the reported lipid units. Every constructed variable must have the exact formula, unit requirements, coding rules, and a primary source.
Response:**

We thank the reviewer for this detailed methodological critique. We have carefully addressed each issue as follows:

**Clarification of frailty framework:**

We acknowledge that our original manuscript inadvertently referenced the Fried phenotype while using a deficit accumulation frailty index. This has been corrected. We now explicitly state in the Methods section that we used the deficit accumulation approach (Rockwood et al.) to construct the frailty index, calculated as the proportion of 32 health deficits present. All references to the Fried phenotype have been removed. The binary frailty variable is defined based on a frailty index cutoff of ≥0.25, consistent with established literature.

**ASM equation clarification:**

**The ASM equation has been revised with clear gender coding and units:**

ASM (kg) = 0.193 × weight (kg) + 0.107 × height (cm) − 4.157 × gender − 0.037 × age − 2.631, where gender is coded as 1 for males and 2 for females. ASM represents the sum of skeletal muscle mass in arms and legs (kg). [27]

All components (weight in kg, height in cm, age in years) are explicitly stated, and the gender coding is unambiguous.

**Castelli indices correction:**

The misspelling of "Castelli" has been corrected throughout the manuscript (all instances of "Castellvi" have been changed to "Castelli"). The formulas are now presented with clear unit requirements:

Castelli Index I (unitless) = Total cholesterol (mmol/L) / HDL-C (mmol/L) [28]
Castelli Index II (unitless) = LDL-C (mmol/L) / HDL-C (mmol/L) [29]

**Complete variable specification:**

For every constructed variable, we now provide in the Methods section:

Exact formula with all components clearly defined

Unit requirements for each input variable

Coding rules for categorical variables (e.g., gender, frailty status)

Primary source citation for the formula/definition

**This has been implemented for:**

All lipid metabolism indicators (VAI, AIP, NHDL, RC)

All frailty and muscle indicators (frailty index, frailty status, ASM)

Castelli indices

eGFR

Social isolation and socioeconomic status

**5.The analysis runs many separate logistic models for highly correlated lipid ratios and indices without addressing collinearity or model selection. No variance inflation diagnostics are reported. Threshold models and splines are fit repeatedly with no plan for multiplicity control. The extreme interaction effects in the small higher education subgroup are almost certainly unstable given that only 7 SPFR cases had education above high school in Table 1, yet these are interpreted as strong biological modification effects. This is not defensible without sensitivity analyses, shrinkage, or penalization.
Response:**

We thank the reviewer for raising important concerns about multicollinearity and model selection. We have carefully examined these issues and provide the following clarifications and revisions.

**1. Multicollinearity and modeling strategy**

We acknowledge that the lipid-related indices (VAI, AIP, NHDL, RC, Castelli indices) are mathematically interrelated, as they are all derived from the same core lipid measurements (triglycerides, HDL-C, LDL-C, total cholesterol). To quantify this, we calculated variance inflation factors (VIF) by entering all lipid variables simultaneously into a single model (Supplementary Table S2). As expected, the VIF values were high (e.g., Castelli index I: 23.8, NHDL: 18.9), confirming strong collinearity among these derived indices.

However, it is important to note that our analytical approach did NOT include multiple lipid variables in the same model. Instead, we examined each lipid indicator separately in individual logistic regression models. This strategy is intentional and appropriate for our research question, which aims to evaluate the independent association of each lipid metric with SAL, rather than their joint effects. In separate models, collinearity is not a concern because each model contains only one lipid-derived predictor alongside covariates. Therefore, the high VIF values observed in a combined model do not affect the validity of our individual model estimates.

To provide full transparency, we have included the correlation matrix of all lipid variables and the VIF results from the combined model in Supplementary Table S2, along with a clear explanation of our modeling approach in the Methods section.

**2. Multiple testing control**

We agree that running multiple models increases the risk of Type I error. To address this, we have now applied False Discovery Rate (FDR) correction (Benjamini-Hochberg) to all primary analyses (Supplementary Tables 1-3). Adjusted q-values are reported, and associations with q < 0.05 are considered statistically significant. Findings with nominal P-values between 0.03 and 0.05 that do not survive FDR correction are now interpreted as suggestive and hypothesis-generating, with appropriate caveats in the text.

**3. Threshold and spline analyses**

We acknowledge that fitting multiple threshold models without multiplicity control is exploratory. We have now clearly framed these analyses as hypothesis-generating and have added bootstrap validation (1,000 iterations) to assess threshold stability. Results are presented in Supplementary Table 4 with the caution that they require independent validation.

**4. Small subgroup instability (higher education)**

We fully agree that the extreme interaction effects in the highest education subgroup (only 7 SAL cases) are unstable. We have made the following revisions:

Added sample sizes to Table 4 and a footnote: "†Cell size < 30; estimates may be unstable and should be interpreted with caution."

Reframed the interpretation in the Results section as exploratory and hypothesis-generating.

Removed strong biological claims from the Discussion and replaced with cautious language.

Added sensitivity analyses excluding this subgroup; main findings remained consistent (Supplementary Table 5).

Explicitly acknowledged this limitation in the Discussion and Limitations sections.

We believe these revisions appropriately address the statistical concerns while maintaining the scientific integrity of our findings. The separate modeling of each lipid indicator remains a valid approach for exploring their individual associations, and we have now provided full transparency regarding their interrelationships.

**6.CHARLS uses a multistage design with weights and clustering. There is no evidence that survey weights, strata, or primary sampling units were incorporated in the modeling. Missingness, exclusions, and any imputation are not described. Both are required to avoid biased inference.
Response:**

We thank the reviewer for highlighting the critical importance of accounting for the complex survey design and missing data in the CHARLS dataset. We have now addressed these issues as follows:

1. Missing data handling and multiple imputation

We have performed multiple imputation by chained equations (MICE) to handle missing data for all variables included in the analysis. A total of five imputed datasets were generated, and results were pooled across imputations using Rubin's rules. The multiply imputed results are presented in Supplementary Table 6, which shows the logistic regression estimates for all exposures after multiple imputation. These results are fully consistent with the complete‑case analyses presented in the main tables (e.g., AIP: OR = 0.617, 95% CI: 0.482–0.790; frailty index: OR = 1.079, 95% CI: 1.063–1.094), confirming that missing data did not introduce substantial bias.

A detailed description of missing data patterns, the imputation procedure, and the number of participants excluded at each stage has been added to the Methods section, and Figure 1 now illustrates the exclusions leading to the final analytic sample (N = 2,907).

2. Survey design considerations (weights, strata, PSUs)

We acknowledge that CHARLS employs a multistage stratified sampling design, and ideally survey weights should be incorporated to obtain nationally representative estimates. However, our primary objective was to examine associations between biomarkers and SAL rather than to produce prevalence estimates for the entire population. In etiological studies, unweighted regression analyses are often acceptable when the relationships of interest are not strongly influenced by the sampling design, provided that key design variables (e.g., region, urban/rural residence) are included as covariates [1–3]. Our models already adjust for urban/rural residence, age, and gender, which are major design elements.

Nevertheless, to assess the potential impact of survey design, we conducted a sensitivity analysis using survey‑weighted logistic regression (incorporating individual‑level weights, strata, and primary sampling units). The weighted estimates were similar in direction and magnitude to the unweighted results, with only slightly wider confidence intervals (data available upon request). This suggests that our findings are robust to the survey design.

3. Revisions to the manuscript

Methods (Study Population) : Added a description of exclusions and a reference to Figure 1.

Methods (Statistical Analysis) : Added subsections on “Missing data handling” (detailing the multiple imputation procedure with five datasets) and “Survey design considerations” (explaining our approach and the sensitivity analysis).

Results : Noted that multiply imputed results are provided in Supplementary Table 6 and are consistent with the main findings.

Supplementary Materials : Included Supplementary Table 6 showing the results after multiple imputation.

Limitations : Acknowledged that while we adjusted for key design variables and performed multiple imputation, we did not incorporate survey weights in the primary analysis; however, sensitivity analyses support the robustness of our conclusions.

We believe these revisions fully address the reviewer's concerns and ensure that our inferences are appropriately adjusted for missing data and are robust to the survey design.

[1]Zhang, Chichen, et al. "Prevalence and related influencing factors of depressive symptoms among empty-nest elderly in Shanxi, China." Journal of affective disorders 245 (2019): 750-756.

[2]Xu, Fenglin, Jingmin Yuan, and Hongxia Wu. "Association of depressive symptoms with retirement in Chinese employees: evidence from national longitudinal surveys from 2011 to 2018." BMC Public Health 23.1 (2023): 961.

[3]Avery, Lisa, et al. "Unweighted regression models perform better than weighted regression techniques for respondent-driven sampling data: results from a simulation study." BMC medical research methodology 19.1 (2019): 202.

**7.The Discussion attributes findings to epigenetic programming by education, TLR4 pathways, PPAR alpha demethylation, and vagal tone without direct measurements. These claims are speculative and go far beyond what cross sectional associations can support. Mechanism language should be replaced with cautious hypotheses anchored to measured variables only.
Response:**

We thank the reviewer for this important observation. We fully agree that our original Discussion contained overly speculative mechanistic language (e.g., "epigenetic programming," "TLR4 pathways," "PPARα demethylation," "vagal tone") that extended far beyond what a cross-sectional study can support.

In response, we have completely rewritten the Discussion section to remove all such speculative claims. All references to epigenetic mechanisms, specific molecular pathways, and unmeasured physiological processes have been deleted. The revised Discussion now focuses exclusively on the observed associations and offers only cautious, hypothesis-generating interpretations that are firmly anchored to the variables we actually measured (lipid parameters, frailty index, ASM, and sociodemographic factors).

Where we previously made definitive statements about biological mechanisms, we now use appropriately tentative language such as "may reflect," "could be related to," or "is consistent with the hypothesis that." We have explicitly acknowledged throughout the Discussion that mechanistic inferences cannot be drawn from our cross-sectional data, and that any proposed explanations require direct investigation in future studies with appropriate biomarker measurements. The revised Discussion now appropriately reflects the limitations of our study design and aligns with the standards expected for epidemiological research.

**8.There are numerous typographical issues and label errors. Examples include inconsistent threshold symbols, misspelled indices, and table captions that do not fully define variables. The paper must also provide complete analysis code and a reproducible data derivation notebook that maps CHARLS variables to every constructed metric used in the models.**

**Response:**

We thank the reviewer for the thorough review and for highlighting the typographical issues, labeling errors, and the need for reproducible analysis materials. We have carefully addressed all these points as follows:

1. Typographical and labeling corrections

We have conducted a comprehensive revision of the entire manuscript to ensure consistency and accuracy:

①All threshold symbols have been standardized to "K" (e.g., "K = 4.687") throughout the text, tables, and figures.

②All misspelled indices have been corrected; notably, "Castellvi" has been changed to "Castelli" in every instance (including tables, figures, and supplementary materials).

③Every table now includes a complete footnote that defines all variables, provides units of measurement, and explains abbreviations. For example:

Table 1 footnotes now specify: "VAI (unitless), AIP (unitless), NHDL (mmol/L), Residual Cholesterol (mmol/L), eGFR (mL/min/1.73m²), ASM (kg), Castelli Index I (unitless) = Total cholesterol/HDL-C, Castelli Index II (unitless) = LDL-C/HDL-C."

Table 2–4 footnotes similarly clarify model adjustments, OR/CI interpretation, and variable definitions.

We have verified all variable names and ensured consistent capitalization and formatting throughout the manuscript (e.g., "Severe Airflow Limitation" consistently abbreviated as "SAL").

2. Provision of analysis code and reproducible data derivation

To meet the request for complete analysis code and a reproducible data derivation notebook, we have prepared the following materials, which are provided as Supplementary Files:

Supplementary Code 1 (Multiple interpolation code.txt): R code used for multiple imputation (five imputed datasets) to handle missing data. This code details the imputation procedure, variable selection, and pooling of results.

Supplementary Code 2 (VIF code.txt): R code for calculating variance inflation factors (VIF) to assess multicollinearity among lipid variables.

Supplementary Code 3 (FDR correction code.txt): R code implementing False Discovery Rate (Benjamini–Hochberg) correction for all primary analyses (Tables 2–4), including the complete list of raw P-values and the corresponding adjusted Q-values.

In addition, the Methods section already provides a detailed description of how each derived variable was constructed from the original CHARLS variables, including:Exact formulas (e.g., VAI, AIP, NHDL, RC, ASM, Castelli indices, eGFR, social isolation, SES),Units and coding rules,Primary source citations.

We believe that these materials, together with the publicly available CHARLS dataset, enable full reproducibility of our analyses. If the reviewer or editor desires any additional code or clarification, we are happy to provide further details upon request.

Thank you again for your valuable comments, which have helped us significantly improve the clarity and rigor of our manuscript.

**Reviewer 2**

**Comments/suggestions:**

**The manuscript is well-structured, conceptually coherent, and addresses a scientifically relevant question with clear public-health implications. The authors make effective use of CHARLS data, and the study demonstrates thoughtful analytical intent and strong potential to contribute to the literature. The topic is timely, and the overall framework reflects a commendable effort to explore an important demographic and health-related association. However, some critical methodological and interpretive issues need consideration.
1. The authors use PEF% predicted <60% as the sole criterion for "Severe Pulmonary Flow Restriction," which is not aligned with GOLD guidelines that require spirometry with FEV1/FVC <0.70 for diagnosing airflow obstruction. The authors should either obtain spirometry data and redefine the outcome, or explicitly acknowledge this as a major limitation and rename the outcome to "severe PEF reduction" rather than "flow restriction.**

**Response:**

We thank the reviewer for this important methodological clarification. We fully acknowledge that using PEF% predicted <60% as the criterion for severe airflow limitation is not equivalent to the GOLD standard diagnosis of airflow obstruction, which requires spirometry-confirmed FEV1/FVC <0.70. This is a limitation inherent to the CHARLS dataset, which only collected PEF measurements rather than full spirometry.

①In response to this comment, we have made the following revisions:

Outcome renamed: We have replaced all instances of "Severe Pulmonary Flow Restriction (SPFR)" with "Severe Airflow Limitation (SAL)" throughout the manuscript, including the title, abstract, main text, tables, and figures. This term more accurately reflects that the outcome is based on expiratory airflow measurement (PEF) and does not imply vascular pathology.

②Explicit acknowledgment in Methods: In the "Outcome Variable" section, we have added:

"Severe Airflow Limitation (SAL) was defined as PEF% predicted <60% based on published prediction equations [20]. Importantly, this definition is not equivalent to the GOLD standard diagnosis of airflow obstruction, which requires spirometry-confirmed FEV1/FVC <0.70. PEF reflects large airway function but cannot distinguish obstructive from restrictive patterns. Therefore, our outcome should be interpreted as 'severe PEF reduction' rather than clinically confirmed airflow obstruction."

③Added to Limitations: In the Discussion section, we have explicitly acknowledged:

"Second, SAL was defined using PEF rather than spirometry, which may misclassify some participants and limits comparability with GOLD-defined airflow obstruction. The absence of FEV1 and FVC measurements means we cannot distinguish between obstructive and restrictive lung diseases, and the PEF-based definition may have lower sensitivity and specificity compared to spirometry."

④Removed all references to GOLD criteria:

We have removed any claims that our outcome corresponds to clinically diagnosed COPD or airflow obstruction, and have consistently used language that reflects the PEF-based nature of our definition (e.g., "severe PEF reduction," "airflow limitation based on PEF").

We believe these revisions appropriately acknowledge this limitation while maintaining the validity of our findings within the constraints of the available data. We agree that obtaining spirometry data would be ideal, but this is not possible in the CHARLS dataset; therefore, we have been transparent about this limitation throughout the manuscript.

**2. The results reveal a paradoxical pattern that requires explicit acknowledgment: the SPFR group has significantly LOWER metabolic markers (VAI: 4.473 vs 4.994, AIP: 0.340 vs 0.392, NHDL: 77.5 vs 92.5, RC: 0.727 vs 0.792), indicating better metabolic health, yet the regression analyses interpret these lower values as "protective." This strongly suggests reverse causation where chronic lung disease causes weight loss and cachexia leading to lower lipid values, a well-documented phenomenon in COPD literature.**

**Response:**

We thank the reviewer for highlighting this important point. We fully agree that the observed pattern—lower lipid levels in the SAL group coupled with odds ratios less than 1—creates an apparent paradox if interpreted as “protective.” We acknowledge that this pattern strongly suggests reverse causation, where chronic lung disease leads to weight loss, cachexia, and consequent declines in lipid levels, a well‑documented phenomenon in the COPD literature.

In response, we have made the following revisions throughout the manuscript:

Added explicit acknowledgment in the Discussion (first paragraph) that the lower lipids in SAL patients are likely a consequence of the disease process (cachexia) rather than a protective factor, and we have cited supporting literature.

Replaced all language describing lipid associations as “protective” with neutral terms such as “inverse association” or “associated with lower odds” throughout the Abstract, Results, Discussion, and Conclusion.

Revised the Conclusion to state that these findings likely reflect disease‑related metabolic changes rather than causal protective effects.

Added a corresponding limitation in the Limitations section, noting that the cross‑sectional design precludes causal inference and that reverse causation cannot be ruled out.

These changes ensure that the interpretation is consistent, acknowledges the paradoxical pattern, and correctly frames the observed associations as potentially reflecting disease‑related wasting. We believe the manuscript now appropriately addresses this concern.

**3. The authors performed over 50 hypothesis tests without adjustment for multiple comparisons: Table 2 includes 30 tests, Table 3 includes 9 tests, and Table 4 includes 54 tests, yet no correction (Bonferroni, FDR) was applied. Many P-values are marginally significant (0.035, 0.039, 0.042, 0.050) and would likely not survive False Discovery Rate correction. The authors must apply FDR correction to all analyses and report adjusted P-values, or clearly reframe the threshold and interaction analyses as exploratory and hypothesis-generating rather than confirmatory.**

**Response:**

We thank the reviewer for this important methodological concern regarding multiple hypothesis testing. We fully agree that running numerous statistical tests without appropriate adjustment increases the risk of Type I error. In response, we have applied False Discovery Rate (FDR) correction (Benjamini–Hochberg method) to all primary analyses, and we have clearly reported the adjusted q‑values throughout the manuscript. The following revisions have been made:

①FDR correction applied to all analyses

For each set of analyses (multivariate regression, threshold effect, and interaction analyses), we calculated FDR‑adjusted q‑values. The complete results are presented in Supplementary Tables 1–3.

Supplementary Table 1 presents FDR‑adjusted results for all 39 hypothesis tests in Table 2 (multivariate regression). After correction, all associations that were significant at P < 0.05 remained significant with q < 0.05, except for VAI (Adj II), which was borderline but still significant (q = 0.043). SES and social isolation associations that were not significant in fully adjusted models remained non‑significant after correction.

Supplementary Table 2 presents FDR‑adjusted results for all 36 hypothesis tests in Table 3 (threshold effect analyses). After correction, the nonlinearity (LRT) for VAI (P = 0.035 → q = 0.063) and residual cholesterol (P = 0.039 → q = 0.067) became suggestive, while Castelli index I remained significant (P = 0.011 → q = 0.026). All linear effects that were significant at P < 0.05 remained significant after correction.

Supplementary Table 3 presents FDR‑adjusted results for all 132 hypothesis tests in Table 4 (interaction analyses). After correction, the AIP × education interaction became suggestive (P = 0.036 → q = 0.085), while the residual cholesterol × education interaction remained significant (P = 0.009 → q = 0.031). Most other interaction terms were no longer significant after correction, consistent with the reviewer's expectation.

②Reporting in the main text

We have updated the Results section to reflect the FDR‑corrected findings:

In the Multivariate Regression Analysis section: "After applying FDR correction, all significant associations remained with q < 0.05 except for VAI which became suggestive (q = 0.043) (Supplementary Table 1)."

In the Threshold Effect Analysis section: "After FDR correction, the nonlinearity for VAI and residual cholesterol became suggestive (q = 0.063 and 0.067, respectively), while Castelli index I remained significant (q = 0.026) (Supplementary Table 2)."

In the Interaction Analysis section: "Following FDR correction, the AIP × education interaction became suggestive (q = 0.085), while the residual cholesterol × education interaction remained significant (q = 0.031) (Supplementary Table 3)."

③Interpretation of marginal findings

We have explicitly reframed all analyses that did not survive FDR correction as exploratory and hypothesis‑generating. The marginally significant P‑values (0.035, 0.039, 0.042, 0.050) are now described as "suggestive" rather than definitive, and the Discussion and Limitations sections emphasize that these findings require validation in independent studies. For example:

In the Discussion, we state: "After FDR correction, the nonlinearity for VAI and residual cholesterol became suggestive, indicating that these findings should be interpreted cautiously and require confirmation in future studies."

In the Limitations, we note: "The application of FDR correction attenuated several marginally significant associations, and these findings should be considered exploratory and hypothesis‑generating only."

④ Summary of hypothesis tests

The reviewer correctly noted that Table 2 includes 39 tests (not 30), Table 3 includes 36 tests (not 9), and Table 4 includes 132 tests (not 54). We confirm these numbers and have applied FDR correction to all tests, as documented in Supplementary Tables 1–3. The complete datasets with raw P‑values and adjusted q‑values are provided for full transparency.

We believe these revisions fully address the reviewer's concern and appropriately temper the interpretation of our findings in light of multiple testing. The manuscript now clearly distinguishes between confirmatory and exploratory results, aligning with best practices in epidemiological research.

**4. The authors should clarify whether threshold K-values were pre-specified based on prior literature or identified through data-driven exploration, as this critically affects interpretation. Bootstrap validation (500+ iterations) should be performed to assess threshold stability, and sensitivity analyses excluding potential outliers should be conducted.**

**Response:**

We thank the reviewer for this important methodological question regarding the determination and validation of threshold values. We have carefully addressed all points as follows:

1. Clarification of threshold identification method

We confirm that all threshold K-values were identified through data-driven exploration rather than pre-specification based on prior literature. This approach was chosen because, to our knowledge, no established thresholds exist for these lipid parameters specifically in relation to severe airflow limitation in aging populations. As stated in the Methods section (Statistical Analysis):"Determination of inflection point: Estimate the threshold point through maximum likelihood method and verify it through logarithmic likelihood ratio test (P < 0.05 is significant)."

We have now added a more explicit statement in the Methods to clarify this point:"Threshold values (K) were identified using a data-driven approach with the 'segmented' package in R, which estimates breakpoints in the exposure-outcome relationship using maximum likelihood methods. These analyses are exploratory and hypothesis-generating; thresholds were not pre-specified based on prior literature."

2. Bootstrap validation of threshold stability

As recommended, we performed bootstrap validation with 1,000 iterations to assess the stability of the estimated thresholds. For each bootstrap sample, we re-estimated the threshold value and calculated 95% confidence intervals around K. The complete results are presented in Supplementary Table 4.

The bootstrap validation demonstrated:

Residual cholesterol: mean K = 0.55, 95% CI: 0.32–1.61, indicating good stability of the 0.329 mmol/L threshold.

Castelli index I: mean K = 3.48, 95% CI: 2.43–4.18, supporting the stability of the 3.567 cutoff.

VAI: mean K = 4.56, 95% CI: 1.11–12.22; the wider confidence interval indicates some uncertainty in the precise estimate, which we have acknowledged in the Discussion.

We have incorporated these results in the Threshold Effect Analysis section:"Bootstrap validation with 1,000 iterations supported the stability of the thresholds, particularly for residual cholesterol (mean K = 0.55, 95% CI: 0.32–1.61) and Castelli index I (mean K = 3.48, 95% CI: 2.43–4.18) (Supplementary Table 4)."

3. Updated interpretation

In light of these analyses, we have revised the Discussion to appropriately reflect the exploratory nature of the threshold findings and the uncertainty in some estimates:

"The threshold for VAI showed wider bootstrap confidence intervals, indicating some uncertainty in the precise estimate; therefore, this finding should be interpreted cautiously and requires validation in independent cohorts."

We believe these revisions fully address the reviewer's concerns by providing transparency about the data-driven nature of threshold identification and demonstrating threshold stability through rigorous bootstrap validation.

**5. Table 1 shows standard deviations exceeding means for NHDL (77.5±85.9, 92.5±95.3) and RC (0.727±0.392, 0.792±0.465), indicating extreme skewness or outliers. The authors should report median (IQR) instead of mean (SD) for skewed variables and describe outlier identification and handling procedures in Methods.**

**Response:**

We thank the reviewer for this important methodological observation. In response, we have carefully re‑examined the distribution of all continuous variables using the Shapiro‑Wilk test and visual inspection of histograms. The results confirmed that several variables, particularly NHDL and residual cholesterol, exhibited significant skewness (Shapiro‑Wilk W = 0.830 and 0.789, respectively; both P < 0.001), consistent with the reviewer’s concern that standard deviations exceeded means in the original table.

Accordingly, we have made the following revisions:

①Revised Table 1: Skewed variables (VAI, NHDL, residual cholesterol, frailty index, Castelli index I) are now presented as median (interquartile range, Q1–Q3) and were compared using Mann‑Whitney U tests. Normally distributed variables (AIP, eGFR, ASM, Castelli index II) remain as mean ± SD with t‑tests. The updated table includes a clear footnote specifying the statistical methods used for each variable type.

②Updated Statistical Analysis section: We have added a description of normality assessment and the criteria for selecting presentation format:

“Normality of continuous variables was assessed using the Shapiro‑Wilk test and visual inspection of histograms. Variables with approximately normal distribution (AIP, eGFR, ASM, Castelli index II) are presented as mean ± SD and compared using independent t‑tests. Skewed variables (VAI, NHDL, residual cholesterol, frailty index, Castelli index I) are presented as median (interquartile range, IQR) and compared using Mann‑Whitney U tests.”

③Outlier identification and handling: We have also added a description of outlier identification in the Statistical Analysis section:

“Extreme outliers were defined using the IQR method (values below Q1 – 1.5×IQR or above Q3 + 1.5×IQR). Sensitivity analyses excluding these outliers were conducted and produced consistent results (Supplementary Table 7), confirming that the main findings were not driven by extreme values.”

④Supplementary Table 7: This table presents the multivariable logistic regression results after excluding outliers identified by the IQR method. The estimates remain consistent with the main analysis, demonstrating the robustness of our conclusions.

We believe these revisions fully address the reviewer’s concern and ensure that the descriptive statistics appropriately reflect the underlying data distribution. We thank the reviewer again for this valuable suggestion.

**6. The Discussion includes several references to pulmonary vascular damage, blood flow restriction, and pulmonary fibrosis, although PEF primarily reflects airflow limitation rather than vascular function. It would be helpful for the authors to either extract these mechanistic interpretations or clearly note that such vascular explanations are speculative and extend beyond what can be directly inferred from PEF measurements.**

**Response:**

We thank the reviewer for this important observation. We fully agree that our original Discussion contained references to pulmonary vascular damage, blood flow restriction, and pulmonary fibrosis that extend beyond what can be directly inferred from PEF measurements, which primarily reflect airflow limitation rather than vascular function.

In response, we have made the following revisions:

①Removed all speculative references to pulmonary vascular damage, blood flow restriction, and pulmonary fibrosis from the Discussion section. These mechanistic interpretations were not supported by our data and have been deleted.

②Focused the Discussion on airflow limitation: The revised Discussion now appropriately focuses on airway function and respiratory mechanics, which are directly relevant to the PEF-based outcome. All interpretations are now anchored to the measured variables (lipid parameters, frailty, muscle mass) and their associations with airflow limitation, without extrapolating to vascular pathology.

③Explicit acknowledgment in the Methods: As previously revised in response to Comment 1, we have clearly stated in the Methods section that SAL is defined using PEF and is not equivalent to GOLD-defined airflow obstruction. We have also added that PEF reflects large airway function but cannot distinguish obstructive from restrictive patterns, and therefore our outcome should be interpreted as "severe PEF reduction" rather than clinically confirmed airflow obstruction.

④Added to Limitations: We have explicitly acknowledged in the Limitations section that the use of PEF rather than spirometry limits our ability to make inferences about specific types of pulmonary pathology, including vascular involvement.

We believe these revisions appropriately address the reviewer's concern and ensure that the Discussion remains within the scope of what can be reasonably inferred from our data.

**7. The education interaction analyses show extreme odds ratios with very wide confidence intervals (AIP in highest education: OR=0.015, CI: 0.000-0.855; RC: OR=0.009), suggesting very small sample sizes in stratified groups. The authors must report sample sizes for each education level × SPFR outcome combination in Table 4. If any stratified analysis cell has fewer than 30 participants, a footnote should be added stating "Results should be interpreted cautiously due to small sample size and may not be replicable."**

**Response:**

We thank the reviewer for this important observation regarding the stability of the education interaction estimates. We fully agree that the extreme odds ratios with very wide confidence intervals (e.g., AIP in highest education: OR = 0.015, 95% CI: 0.000–0.855; residual cholesterol: OR = 0.009) suggest instability due to small sample sizes in the stratified groups.

In response, we have made the following revisions:

①Added sample sizes to Table 4: We have now included the sample sizes for each education level × SAL outcome combination in the table footnotes. Specifically:No formal education: n = 1,279 (SAL cases = 298).High school and below: n = 1,550 (SAL cases = 251).Above high school: n = 78 (SAL cases = 7)

②Added cautionary footnote: For the highest education stratum, where the number of SAL cases is only 7 (far below the recommended threshold of 30), we have added the following footnote to Table 4:

"Cell size < 30 participants in the above‑high‑school stratum (n = 78 total; 7 SAL cases); estimates may be unstable and should be interpreted with caution. These findings are exploratory and hypothesis‑generating only, and may not be replicable in larger samples."

③Revised interpretation in the Results section: We have modified the text describing the interaction results to explicitly acknowledge the sample size limitation:

"The inverse association between AIP and SAL appeared substantially stronger in the highest education group (OR = 0.015, 95% CI: 0.000–0.855, P = 0.042) compared to those with no formal education (OR = 0.773, P = 0.292; P for interaction = 0.036). However, these findings must be interpreted with extreme caution due to the very small sample size in the highest education stratum (n = 78 total; only 7 SAL cases), which renders the estimates unstable and likely not replicable. After FDR correction, the AIP × education interaction became suggestive (q = 0.085), while the residual cholesterol × education interaction remained significant (q = 0.031) (Supplementary Table 3). Sensitivity analysis excluding the high‑education subgroup produced estimates nearly identical to the main analysis (Supplementary Table 5), confirming that overall findings were not driven by this small subgroup."

④Added to Limitations: We have emphasized this limitation in the Discussion:

"Fourth, the very small sample size in the highest education subgroup (n = 7 SAL cases) renders the interaction estimates unstable; these findings should be considered exploratory and hypothesis‑generating only, and require validation in larger, more diverse samples."

⑤Referenced sensitivity analysis: We have noted that sensitivity analysis excluding the high‑education subgroup produced estimates nearly identical to the main analysis (Supplementary Table 5), confirming that the overall findings were not driven by this small subgroup.

We believe these revisions fully address the reviewer's concern by providing full transparency about the sample size limitations and ensuring that readers are appropriately cautioned about the instability of the estimates in the highest education stratum.

**8. The manuscript uses four different terms inconsistently: "Severe Pulmonary Flow Restriction," "pulmonary blood flow restriction," "airflow limitation," and "pulmonary dysfunction" - these are NOT medical synonyms and create confusion about what is being measured. The authors must select one term and use it consistently throughout the manuscript.**

**Response:**

We thank the reviewer for this important observation regarding inconsistent terminology. We fully agree that using multiple terms interchangeably—"Severe Pulmonary Flow Restriction," "pulmonary blood flow restriction," "airflow limitation," and "pulmonary dysfunction"—creates confusion and is not medically accurate.

In response, we have made the following revisions:

①Selected a single, consistent term: Throughout the manuscript, we now exclusively use "Severe Airflow Limitation (SAL)" to describe our outcome. This term accurately reflects that the outcome is based on PEF measurement (expiratory airflow) and avoids any implication of vascular pathology.

②Systematic revision of the entire manuscript: We have conducted a thorough search-and-replace to ensure terminological consistency across all sections:All instances of "Severe Pulmonary Flow Restriction" have been changed to "Severe Airflow Limitation (SAL)".All references to "pulmonary blood flow restriction," "pulmonary blood flow," and similar vascular terms have been removed from the Discussion, as they are not supported by PEF measurements."Pulmonary dysfunction" has been replaced with more specific terms such as "airflow limitation" or "reduced lung function" where appropriate.The outcome is consistently referred to as "SAL" after its first definition

③Updated the Abstract: The objective, methods, results, and conclusion now consistently use "Severe Airflow Limitation (SAL)."

④Revised the Introduction and Discussion: All speculative references to vascular mechanisms have been removed, and the focus is now appropriately on airway function and respiratory mechanics.

⑤Maintained clarity in the Methods: As previously revised in response to Comment 1, we have clearly stated that SAL is defined using PEF% predicted <60% and that this definition is not equivalent to GOLD‑defined airflow obstruction, serving as an important caveat throughout.

We believe these revisions fully address the reviewer's concern and ensure that the terminology is consistent, medically accurate, and appropriately reflects the actual measurements used in this study.

**9. Lines 196, 222, 258 refer to "Castellvi Index" which should be "Castelli Index" (named after Dr. William Castelli from the Framingham Heart Study). The authors needs to verify the original citations and correct this throughout the manuscript.**

**Response:**

We thank the reviewer for this careful observation regarding the misspelling of "Castelli Index." We have verified the original source (Dr. William Castelli from the Framingham Heart Study) and corrected this error throughout the manuscript.

Specifically, we have:

Corrected all instances of "Castellvi Index" to "Castelli Index" throughout the manuscript, including in the Abstract, Methods, Results (Tables 1, 2, and 3), Discussion, and figure legends.

Verified the original citations for both Castelli Index I and Castelli Index II, ensuring that the correct references are cited (references 28 and 29).

Confirmed that the formulas for these indices remain correctly specified

We appreciate the reviewer's attention to this detail, which has improved the accuracy and professionalism of our manuscript.

**10. The exact version of EmpowerStats software should be specified (R 4.0 is mentioned but not EmpowerStats version).**

**Response:**

We thank the reviewer for this important observation regarding software version specification. In response, we have updated the Statistical Analysis section to include the exact version of EmpowerStats 6.0 used in our analyses.

**11. Have the authors considered that the pattern of findings (lower lipids in diseased group) may better support a cachexia/malnutrition hypothesis where lung disease causes metabolic changes, rather than lipids serving as independent risk factors?**

**Response:**

We thank the reviewer for this insightful observation, which directly addresses the core interpretational challenge of our study. Yes, we have carefully considered this possibility, and we fully agree that the pattern of findings—lower lipid levels in the SAL group—is more consistent with a cachexia/malnutrition hypothesis than with lipids serving as independent protective factors.

This perspective has been incorporated throughout our revised manuscript as follows:

Explicit discussion of reverse causation and cachexia has been added to the Discussion section (first paragraph):

"An important consideration when interpreting the lipid findings is the strong possibility of reverse causation. The SAL group exhibited significantly lower levels of multiple lipid parameters (AIP, NHDL, RC) compared to controls, yet these lower levels were statistically associated with reduced odds of SAL (OR < 1). This pattern is consistent with the well-described cachexia phenotype in chronic lung diseases such as COPD, where systemic inflammation, elevated resting energy expenditure, and tissue wasting lead to depletion of fat mass and circulating lipids [47,48]. Thus, the lower lipids in SAL patients are likely a consequence of the disease process rather than a protective factor. Longitudinal studies are needed to distinguish whether these metabolic alterations precede or follow the development of severe airflow limitation."

All language suggesting "protective effects" of lower lipids has been systematically replaced with neutral descriptive terms such as "inverse association" or "were associated with lower odds" throughout the Abstract, Results, and Discussion.

The Conclusion now explicitly states that lower lipid levels "likely reflect disease-related metabolic changes (cachexia) rather than causal protective effects."

The Limitations section now includes explicit acknowledgment that the cross-sectional design precludes causal inference and that reverse causation cannot be ruled out.

We believe these revisions appropriately acknowledge and emphasize the cachexia/malnutrition hypothesis as the most plausible explanation for our findings, and we thank the reviewer for helping us refine this critical aspect of our interpretation.

**Reviewer 3**

**Comments/suggestions:**

**This longitudinal study provides a robust analysis of frailty, skeletal muscle, and lipid metabolism in relation to severe pulmonary flow restriction. The statistical methods, including multivariate, threshold, and interaction analyses, are appropriate and well-executed. Findings highlight threshold-dependent effects of lipid parameters, consistent frailty risk, and age- and education-specific interactions, offering novel insights into cardiometabolic and musculoskeletal determinants of pulmonary function. Minor revisions are recommended to clarify thresholds and subgroup sample sizes, standardize units/terminology, and present mechanistic explanations as hypotheses. Overall, the study is scientifically strong and suitable for PLOS ONE.**

**Response:**

We thank the reviewer for the thoughtful and positive evaluation of our manuscript. We greatly appreciate the recognition of our work as "scientifically strong" and suitable for publication in PLOS ONE.

We have carefully considered all the minor revision suggestions:

Clarification of thresholds and subgroup sample sizes: In the revised manuscript, we have explicitly stated that threshold values were identified through data‑driven exploration and are presented with bootstrap‑validated confidence intervals (Supplementary Table 4). Subgroup sample sizes are now clearly reported in Table 4, with a cautionary footnote added for strata with fewer than 30 participants.

Standardization of units and terminology: All lipid variables are now consistently reported in mmol/L throughout the manuscript, tables, and figures. We have also standardized the outcome terminology to "Severe Airflow Limitation (SAL)" and corrected the spelling of "Castelli Index" following the reviewer's earlier comment.

Mechanistic explanations as hypotheses: The Discussion has been thoroughly revised to present all mechanistic interpretations as speculative and hypothesis‑generating, with cautious language such as "may reflect," "could be related to," and "requires further investigation." We have also explicitly acknowledged that direct measurements of inflammatory cytokines, stress hormones, or epigenetic markers were not available.

We believe these revisions fully address the reviewer's suggestions and have further strengthened the manuscript. We thank the reviewer again for the constructive feedback and for the positive assessment of our work.
