# Supplementary material for: Nonlinear thresholds in lipid-frailty interplay: Precision targets for severe airflow limitation in aging adults
Source: PLoS One. 2026 Apr 29;21(4):e0348083. doi: 10.1371/journal.pone.0348083 (PMC13127961; doi:10.1371/journal.pone.0348083)
Supplement: Attachment — Submitted filename: Response_to_Reviewers_auresp_2.docx [file pone.0348083.s022.docx]

**Point-by-point Response Letter-[Manuscript ID:PONE-D-25-40637]**

Title:**Nonlinear Thresholds in Lipid-Frailty Interplay: Precision Targets for Severe Airflow Limitation in Aging Adults**

**Dear Editors and Reviewers of Plos One,**

We are deeply grateful for considering our manuscript for ***Plos One*** and value the insightful comments offered. These suggestions have been crucial for enhancing our manuscript's quality, and we're confident the revisions have significantly improved its clarity, rigor, and impact. We've meticulously revised the manuscript, addressing each comment with careful consideration and research. Besides responding to the feedback, we've also thoroughly reviewed the entire manuscript, making additional refinements to ensure top - notch scientific and literary quality. All changes are clearly tracked in the revised manuscript, and a clean version is also prepared. Below are our detailed point-by-point responses to each comment. We've aimed to be comprehensive and transparent in our explanations, providing evidence and reasoning for the changes made. Thank you again sincerely for your dedication and hard work. Your expertise is invaluable to the scientific community, and we're honored to contribute to this prestigious journal.

Yours Sincerely,

ZhongQiang Guo

Henan University

**[guozhongqiang0701@gmail.com](mailto:guozhongqiang0701@gmail.com)**

**Reviewer 3**

**Comments/suggestions:**

**The authors have made substantial improvements to the manuscript and have adequately addressed the major scientific concerns raised in the initial review. In particular:
Mechanistic statements have been appropriately reframed as hypotheses.
Reverse causation and the lipid paradox are now well discussed.
Subgroup and interaction effects are interpreted cautiously, with sample sizes and sensitivity analyses provided.
Missing data handling is now clearly described.
However, several minor issues remain that require revision before acceptance:
1.Methods: The manuscript still lacks full details on inclusion/exclusion criteria, the number of knots in spline/threshold analyses, and sample sizes within threshold segments.**

**Response:**

We thank the reviewer for this valuable suggestion. We have now added the missing details to the Methods section and Results section to enhance methodological transparency.

1.Inclusion/exclusion criteria: In the Methods section (Study Population), we have added the following text:
 “Participants were included in this analysis if they had complete data on the key exposure variables (lipid parameters, frailty index, and ASM) and the outcome variable (PEF% predicted). Participants with missing data on covariates were handled using multiple imputation as described in the Statistical Analysis section. No additional inclusion or exclusion criteria beyond data availability were applied to maximize sample representativeness.”

2.Spline and threshold analyses: Upon reviewing our analytical approach, we clarified that we did not use restricted cubic splines for threshold identification; instead, we employed piecewise logistic regression using the segmented package in R. We have revised the Methods section (Statistical Analysis) accordingly:
 “To identify potential threshold effects, we fitted piecewise logistic regression models using the segmented package in R. For each continuous exposure, a two-piecewise linear model was fitted, and the optimal breakpoint (threshold, K) was estimated via maximum likelihood methods. Nonlinearity was assessed by comparing the one-line (linear) model with the two-piecewise model using a log-likelihood ratio test (P < 0.05 indicating significant nonlinearity).”
 The description of restricted cubic splines has been removed to avoid methodological confusion.

3.Sample sizes within threshold segments: We have added the sample sizes for each threshold segment to Table 3 (main text). Specifically, we now report the number of participants below and above the estimated threshold for variables with significant nonlinearity (e.g., residual cholesterol, Castelli index I). A footnote has been added to the table to clarify this information.

We believe these additions address the reviewer’s concerns and improve the reproducibility and transparency of our analyses. Thank you again for the careful review.

**2.Results/Tables: Tables 2–4 remain dense and difficult to interpret. Consider moving detailed lipid and ASM subgroup data to supplementary material and simplifying table presentation.**

**Response:**

We thank the reviewer for this constructive suggestion to improve the clarity of our results presentation. In response, we have streamlined Tables 2–4 to highlight the most important findings while moving detailed data to supplementary materials.

Specifically:

Table 2 now presents only the core findings for AIP, residual cholesterol, frailty index, and ASM. Full results for all lipid parameters, muscle indices, and socioeconomic variables are provided in Supplementary Table 8.

Table 3 now focuses on variables with significant nonlinearity (residual cholesterol and Castelli index I). Complete threshold analysis results for all exposures are available in Supplementary Table 9.

Table 4 now displays only the significant education-level interactions (AIP and residual cholesterol). Full interaction results are presented in Supplementary Table 10.

We believe these revisions significantly improve the readability of the main tables while preserving the complete analytical results for interested readers in the supplementary materials. Thank you again for this valuable suggestion.

**3.Figures: Figures should clearly indicate axes, threshold lines, and subgroup comparisons. Visual summaries such as forest plots would improve clarity.**

**Response:**

We thank the reviewer for the suggestion. After careful consideration, we prefer to retain the original figures. Figure 2 already includes clear axis labels and effectively illustrates the nonlinear relationships. Adding threshold lines may over-simplify the continuous nature of these exploratory findings. For subgroup analysis, the current tabular format presents all estimates transparently and is consistent with the main tables. We believe the current figures adequately support the results while maintaining methodological caution. We appreciate the reviewer’s input and are happy to revisit if the editor deems changes necessary.

**4.Baseline Variables: Please clarify why certain baseline variables (e.g., gender, smoking, alcohol use) were not significantly different between groups.**

**Response:**

We thank the reviewer for this insightful observation. We acknowledge that certain baseline variables—namely gender, smoking status, and drinking frequency—did not show statistically significant differences between the SAL and non-SAL groups. We would like to offer several possible explanations for this finding.

First, smoking and alcohol use are known to be strongly associated with lung function decline; however, in cross-sectional studies of older populations, the absence of a significant difference between groups may reflect disease-related behavioral changes. Individuals with severe airflow limitation may reduce or quit smoking and alcohol consumption due to worsening respiratory symptoms, leading to similar current exposure rates between groups. This phenomenon—sometimes referred to as “reverse causation” in behavioral risk factors—has been documented in previous studies of chronic respiratory disease (e.g., COPD patients often reduce smoking intensity after diagnosis).

Second, regarding gender, while some studies report sex differences in COPD prevalence and severity, the lack of a significant difference in our sample may be due to age distribution and survival bias. Our study population consisted of middle-aged and older adults (mean age approximately 62 years), and the higher risk of mortality in males with severe respiratory disease may result in a more balanced sex distribution among survivors. Additionally, the definition of SAL used in this study (PEF% predicted < 60%) may capture a broader spectrum of airflow limitation that is less sex-dependent compared to GOLD-defined COPD.

Third, it is important to note that the absence of significant differences in unadjusted comparisons does not imply a lack of clinical or biological relevance. Multivariate regression analyses—which adjust for potential confounding—are more appropriate for estimating independent associations. In our fully adjusted models, several metabolic and frailty measures remained significantly associated with SAL after controlling for these factors, suggesting that their effects are robust beyond the influence of gender, smoking, and alcohol use.

We have added a brief explanation in the Results section (Baseline Characteristics) to clarify this point, as follows:

“The lack of significant differences in gender, smoking, and alcohol use between groups may reflect disease-related behavioral changes (e.g., smoking cessation or reduced alcohol intake due to respiratory symptoms) and survival bias in this older population. Multivariate analyses were therefore conducted to appropriately adjust for these factors.”

We appreciate the reviewer’s careful attention to detail and hope this explanation addresses the concern.

**5.Abstract: Condensation of the abstract is recommended to highlight key threshold findings and subgroup interactions more succinctly.**

**Response:**

We thank the reviewer for this helpful suggestion. We have condensed the Abstract to highlight the key threshold findings and subgroup interactions more succinctly. Specifically, we have reduced the background and methods descriptions, emphasized the residual cholesterol threshold (0.329 mmol/L) and the education-level interaction effects for AIP and residual cholesterol, and streamlined the conclusion. The revised Abstract now more clearly presents the novel findings of our study. We appreciate the reviewer’s guidance in improving the manuscript’s conciseness.

**6.Minor/Editorial: Ensure consistent terminology (e.g., Castelli Index), correct remaining typographical errors, and standardize P-value and OR formatting throughout.
Overall, the manuscript is scientifically strong and suitable for publication after these minor revisions..**

**Response:**

We thank the reviewer for the careful editorial review. We have thoroughly revised the manuscript to ensure consistent terminology, correct typographical errors, and standardized formatting throughout.

Specifically:

Terminology: We have standardized “Castelli index I” and “Castelli index II” throughout the manuscript (text, tables, and supplementary materials). In the original version, inconsistent forms such as “Castelli Index 1” and “Castelli Index I” appeared; these have been unified.

Typographical errors: We have corrected remaining typos, including “belt” to “below” in education categories, and other minor errors.

P-value formatting: All P-values are now presented as italicized lowercase p (e.g., *P* = 0.001) throughout the text, tables, and figure legends.

OR formatting: Odds ratios and 95% confidence intervals are now consistently presented as “OR (95% CI)” with a space after the comma (e.g., 0.556 (0.394–0.787)).

We have carefully reviewed the entire manuscript to ensure consistency. We appreciate the reviewer’s attention to these details, which have improved the overall quality of the manuscript.
