# Supplementary material for: Nonlinear thresholds in lipid-frailty interplay: Precision targets for severe airflow limitation in aging adults
Source: PLoS One. 2026 Apr 29;21(4):e0348083. doi: 10.1371/journal.pone.0348083 (PMC13127961; doi:10.1371/journal.pone.0348083)
Supplement: Attachment — Submitted filename: Reviewer Comments fro SPFR_PLOS ONE.pdf [file pone.0348083.s017.pdf]

Reviewer Comments on "Nonlinear Thresholds in Lipid-Frailty Interplay: Precision Targets for Pulmonary Flow Restriction in Aging Adult"

This longitudinal study by Deng et al., provides a rigorous and comprehensive analysis of the interplay between frailty, skeletal muscle mass, lipid metabolism, and severe pulmonary flow restriction (SPFR) in a large adult cohort. The study design, statistical analyses—including multivariate regression, threshold modeling, and interaction analyses—are appropriate and support the conclusions. The findings are novel, highlighting threshold-dependent effects of lipid parameters, age- and education-specific interactions, and the central role of frailty and muscle mass in pulmonary health.

Key Strengths:

- Strong mechanistic integration of lipid metabolism, frailty, muscle indices, and social determinants.
- Advanced statistical approach including nonlinear and interaction analyses.
- Subgroup and threshold analyses add biological and clinical insight, particularly for aging and lifestyle-related diseases.

The areas that need improvement on are as below:

Title / Abstract

⇒ Title is appropriate; consider briefly clarifying "severe pulmonary flow restriction (SPFR)" for general readers.

⇒ Abstract is detailed but dense; highlighting key threshold findings and subgroup interactions more succinctly will improve clarity.

Introduction

⇒ Background on lipid metabolism, frailty, and pulmonary function is strong. Mechanistic statements (e.g., TLR4 activation) should be framed as hypotheses.

Methods

⇒ Provide more details on inclusion/exclusion criteria and how SPFR was diagnosed.

⇒ ASM, Castellvi indices, and lipid parameters are well-described; consider moving formulas to supplement. Specify units for all biochemical measures.

⇒ A summary table of covariates (age, gender, education, smoking, alcohol, social isolation, SES) would help.

⇒ Statistical analyses are appropriate; clarify missing data handling, number of knots in spline/threshold analyses, and sample sizes in each segment. A schematic for interaction models could help.

Results

⇒ Baseline tables are dense; consider moving detailed lipid and ASM subgroups to supplement. Clarify why gender, smoking, and drinking were not significantly different at baseline.

- ⇒ Multivariate regression: ensure ORs/95% CIs formatting consistency; highlight which variables remain significant after full adjustment.
- ⇒ Threshold analysis: clarify sample sizes below/above inflection points. Figures (e.g., Figure 2) should clearly label axes and thresholds.
- ⇒ Subgroup analyses are compelling; forest plots could help visualize differences. Discuss extreme ORs in small subgroups cautiously.
- ⇒ Interaction analysis: consider moving rare strata to supplement. Interpret very large effect sizes (e.g., AIP in university education) carefully.

Discussion

- ⇒ Strength: good integration of lipid metabolism, frailty, muscle loss, and social determinants.
- ⇒ Mechanistic explanations (PPARα, TLR4, omega-3, acetaldehyde) should be clearly stated as hypotheses.
- ⇒ Discuss paradoxical findings (residual cholesterol, eGFR, rural vs urban differences) as hypothesis-generating.
- ⇒ Education effect on metabolism is interesting; include sample sizes to contextualize.

Figures / Tables

- ⇒ Figure 2: ensure clear labels and threshold lines.
- ⇒ Figure 3: forest plots or heatmaps could improve clarity.
- ⇒ Tables 2–4 are dense; consider supplemental tables.

Minor / Editorial

- ⇒ Standardize "Castellis Index" vs. "Castelli Index."
- ⇒ Correct typographical errors ("SIUGGEST," "CNA").
- ⇒ Ensure consistent P-value/OR formatting.
- ⇒ Reduce repetition of methods in results.

Overall Recommendation

- ⇒ Suitability for PLOS ONE: Yes, after minor-to-moderate revisions.
- ⇒ Key revisions: clarify thresholds and subgroup sizes, reframe mechanistic statements as hypotheses, improve figure/table readability, standardize units and terminology.
- ⇒ Scientific merit: High. Novel integration of lipid metabolism, frailty, muscle, and social determinants in SPFR. The dataset is robust, analyses well-done, longitudinal design supports inference.
