# Supplementary material for: Nonlinear thresholds in lipid-frailty interplay: Precision targets for severe airflow limitation in aging adults
Source: PLoS One. 2026 Apr 29;21(4):e0348083. doi: 10.1371/journal.pone.0348083 (PMC13127961; doi:10.1371/journal.pone.0348083)
Supplement: Attachment — Submitted filename: Reviewer comments for the revised version.pdf [file pone.0348083.s019.pdf]

Follow-Up Reviewer Comments:

The authors have made substantial improvements to the manuscript and have adequately addressed the major scientific concerns raised in the initial review. In particular:

- Mechanistic statements have been appropriately reframed as hypotheses.
- Reverse causation and the lipid paradox are now well discussed.
- Subgroup and interaction effects are interpreted cautiously, with sample sizes and sensitivity analyses provided.
- Missing data handling is now clearly described.

However, several minor issues remain that require revision before acceptance:

1. **Methods:** The manuscript still lacks full details on inclusion/exclusion criteria, the number of knots in spline/threshold analyses, and sample sizes within threshold segments.
2. **Results/Tables:** Tables 2–4 remain dense and difficult to interpret. Consider moving detailed lipid and ASM subgroup data to supplementary material and simplifying table presentation.
3. **Figures:** Figures should clearly indicate axes, threshold lines, and subgroup comparisons. Visual summaries such as forest plots would improve clarity.
4. **Baseline Variables:** Please clarify why certain baseline variables (e.g., gender, smoking, alcohol use) were not significantly different between groups.
5. **Abstract:** Condensation of the abstract is recommended to highlight key threshold findings and subgroup interactions more succinctly.
6. **Minor/Editorial:** Ensure consistent terminology (e.g., Castelli Index), correct remaining typographical errors, and standardize P-value and OR formatting throughout.

Overall, the manuscript is scientifically strong and suitable for publication **after these minor revisions**.
